# Epimedium‐Curculigo herb pair enhances bone repair with infected bone defects and regulates osteoblasts through LncRNA MALAT1/miR‐34a‐5p/SMAD2 axis

**DOI:** 10.1111/jcmm.18527

**Published:** 2024-07-10

**Authors:** Maomao Miao, Mengying Li, Yunjie Sheng, Peijian Tong, Yang Zhang, Dan Shou

**Affiliations:** ^1^ School of Pharmaceutical Sciences Zhejiang Chinese Medical University Hangzhou China; ^2^ Institute of Orthopeadics and Traumatology The First Affiliated Hospital of Zhejiang Chinese Medical University (Zhejiang Provincial Hospital of Chinese Medicine) Hangzhou China

**Keywords:** Epimedium‐Curculigo herb pair, infected bone defects, LncRNA MALAT1, miR‐34a‐5p, SMAD2

## Abstract

Infected bone defects (IBDs) are the common condition in the clinical practice of orthopaedics. Although surgery and anti‐infective medicine are the firstly chosen treatments, in many cases, patients experience a prolonged bone union process after anti‐infective treatment. Epimedium‐Curculigo herb pair (ECP) has been proved to be effective for bone repair. However, the mechanisms of ECP in IBDs are insufficiency. In this study, Effect of ECP in IBDs was verified by micro‐CT and histological examination. Qualitative and quantitative analysis of the main components in ECP containing medicated serum (ECP‐CS) were performed. The network pharmacological approaches were then applied to predict potential pathways for ECP associated with bone repair. In addition, the mechanism of ECP regulating LncRNA MALAT1/miRNA‐34a‐5p/SMAD2 signalling axis was evaluated by molecular biology experiments. In vivo experiments indicated that ECP could significantly promote bone repair. The results of the chemical components analysis and the pathway identification revealed that TGF‐β signalling pathway was related to ECP. The results of in vitro experiments indicated that ECP‐CS could reverse the damage caused by LPS through inhibiting the expressions of LncRNA MALAT1 and SMAD2, and improving the expressions of miR‐34a‐5p, ALP, RUNX2 and Collagen type І in osteoblasts significantly. This research showed that ECP could regulate the TGF‐β/SMADs signalling pathway to promote bone repair. Meanwhile, ECP could alleviate LPS‐induced bone loss by modulating the signalling axis of LncRNA MALAT1/miRNA‐34a‐5p/ SMAD2 in IBDs.

## 1 INTRODUCTION

The infected bone defects (IBDs) are known as one of the most difficult intractable diseases in clinical orthopaedics,^1^, ^2^ which are accompanied by aberrant bone formation, progressive bone destruction and systemic inflammatory response.^3^, ^4^ According to the epidemiological studies of IBDs, the prevalence of IBDs induced by bone fracture is 20%–30%, the incidence of postoperative infection bone defects is 24%, and the rate of combination with antibiotics is still 4.5%.^5^ Surgical intervention and the placement of vancomycin‐calcium sulphate beads (VCS) are the most commonly used therapies. While delayed union is still a mystery, some reports suggest that some patients experience a long bone union process, a delayed union or nonunion after anti‐infection treatment of local VCS placement.^6^ Hence, effective treatment options for IBDs shall be found quickly.

On basis of the theory of ‘Kidney Governing Bones’ in TCM, Chinese herbal medicines for tonifying kidney and strengthening bone have been applied in clinical treatment of bone repair,^7^ especially *Epimedium brevicornum* maxim and *Curculigo orchioides* Gaertn. The findings suggested that the main active substances of Epimedium were flavonoids, such as Icariin, Epimedin A/B/C, Baohuoside I and so on, which could regulate the activities of osteoblasts, accelerate the fracture healing and promote the balance of bone metabolism.^8^, ^9^, ^10^ The main effective substances of Curculigo orchioides, such as Curculigoside, Orcinol glucoside, Lignan, Curculigo words polysaccharide, could significantly promote the proliferation and alkaline phosphatase activities of MC3T3‐E1 and boost bone formation.^11^, ^12^ Clinical trials have found that application of the herb pair of *Epimedium brevicornum* maxim and *Curculigo orchioides* Gaertn (ECP) could enhance bone repair in some metabolic bone diseases, by alleviating the oxidative damage of H_2_O_2_ on osteoblasts through PI3K/AKT pathway^13^ or AKT/Nrf2/ HO‐1 pathway.^14^ However, the mechanisms and effective ingredients of ECP in IBDs are still unclear.

Previous studies have found that TGF‐β/SMADs signalling pathway played a pivotal role in osteoblast lineage commitment and differentiation, skeletal development and homeostasis. This pathway involved the TGF‐β receptor, a heterodimeric complex consisting of type I and type II receptors on the plasma membrane. Activation of this receptor leaded to the phosphorylation of SMAD2 and SMAD3 transcription factors, which transduce the signal to the nucleus, ultimately triggering gene transcription.^15^ Moreover, some of non‐coding RNAs (ncRNAs) were confirmed to be upstream regulators of TGF‐β/SMADs signalling pathway,^16^ including miR‐34a‐5p, which associated with an inflammatory response in osteoblasts. Our group has previously demonstrated that the miR‐34a‐5p/SMAD2 signalling axis can enhances bone formation in infected bone nonunion models and attenuates lipopolysaccharide‐induced osteoblast inhibition.^17^ Researches showed that Long non‐coding RNAs could competitively bind to miRNAs, indirectly regulating the expression of miRNA target genes due to their sponge‐like effect. Among these, LncRNA Metastasis Associated in Lung Denocarcinoma Transcript 1 (LncRNA MALAT1), one of the few highly conserved nuclear lncRNAs, has been proved to regulate a variety of physiological processes. LncRNA MALAT1 can not only act as a sponge of miR‐34a‐5p in cancer cells,^18^ but also enhance osteoblasts' activities in osteoporosis mice by mediating the miR‐34c/SATB2 axis.^19^ However, the mechanisms of whether LncRNA MALAT1 regulates osteoblast proliferation and differentiation in infected bone defects and how ECP regulates LncRNA MALAT1 in IBDs are still unknown.

In this research, UPLC‐QTRAP‐MS/MS approach was employed to analyse the absorbed constituents, possible targets for bone repair after anti‐infective therapy were predicted with the network pharmacology approach, and molecular biology experimental techniques were used to confirm the mechanism of ECP in IBDs. Thus, this study elucidated the metabolic patterns of ECP in vivo, the mechanisms in vitro and provided a primordial complementary remedy for bone remodelling in IBDs.

## 2 MATERIALS AND METHODS

### 2.1 Establishment of IBDs model

Healthy New Zealand white male rabbits (3 months, 3.0–3.5 kg weight) were punched a 2 mm‐diameter hole in the tibia and sealed with sterile bone wax to establish the bone defects model. Then, the holes were injected with 1 × 10^6^ CFU/mL *Staphylococcus aureus* suspension (China General Microbiological Culture Collection Center, CGMCC) to prepare the IBDs models.^20^ At the end of 4‐week infection, it was diagnosed that 90% rabbits suffer from infected bone defects. The inactivated, necrotic tissue and dead bone in the wound were thoroughly scraped away. As well, the vancomycin‐calcium sulphate (Van‐CS) were prepared by mixing calcium sulphate powder, vancomycin and normal saline in a mixing bowl at a weight ratio of 9.5:1:3.

All of the IBDs models were divided into Model group, Van‐CS group and Van‐CS + ECP group. The Control group were healthy New Zealand white male rabbits. There were 10 animals in each group. The rabbits in the Van‐CS and Van‐CS + ECP groups received the implantation of Van‐CS in the bone defects. After 4‐week anti‐infection, rabbits in Van‐CS + ECP group received ECP intragastrically at 3.34 g/kg/day for 8 weeks. Housed in individual cages, all of the animals were fed in the same way. This study was carried out in the Animal Experiment Center of Zhejiang Academy of TCM (Hangzhou, China, SCXK 2019‐0010).

### 2.2 Effective evaluation of ECP on bone repair

Following an eight‐week treatment period with ECP, the rabbits were humanely euthanized by injecting sodium pentobarbital excessively and the tibias were removed for subsequent experiments.

The tibia specimens underwent examination by micro computed tomography (micro‐CT, SkyScan‐1172, Bruker, Switzerland). For all scans, the tibia specimens were wrapped in cling film to maintain tissue hydration. The femurs were scanned individually, placed securely within a custom holder. Each scan was conducted with a nominal pixel size of 9 μm, utilizing a 0.5 mm Al filter at a voltage of 60 kV and a current of 167 μA, with an entrance exposure time of 590 ms. An increment of 0.4° was used for each angular step, averaging two frames for increased accuracy. Following standardized reconstruction using NRecon software, the datasets were analysed using CTAn software (Bruker micro‐CT, Belgium). The indexes of bone mineral density (BMD) and bone volume fraction (BV/TV) were calculated.

Then, embedded with paraffin, the tibia specimens were cut into 5 μm slices. After treating with 3% H_2_O_2_ and 5% BSA, slices were added with anti‐RUNX2 (1:200, bs‐1134R, Bioss) at 4°C overnight individually and used for immunohistochemical analysis according to the routine protocol. The expressions of RUNX2 were observed by microscope (Nikon Eclipse Ti, 531,608).

### 2.3 Preparation of ECP extract and ECP‐CS

*Epimedium brevicornum* maxim and *Curculigo orchioides* Gaertn (Zhejiang Chinese Medical University Chinese Medicine Slices Co., Ltd, Lot 220,701) were mixed in a weight proportion of 1:1, based on clinical experience. The mixed materials were subjected to an overnight soaking in 10 × distilled water (V/W) overnight, followed by two rounds of boiling at 100°C 1 hour each time and concentrated to 7.4 g/mL. The ECP extract was stored at −20°C before using.

Sprague–Dawley rats (male, body weight 200 ± 20 g) were randomly fallen into Control group and ECP group with 15 rats in each group. The rats in ECP group were given ECP extract at dose of 7.4 g/kg/day. Following the last gavage, blood sample was extracted from the abdominal aorta. Finally, the ECP containing medicated serum (ECP‐CS) was filtered through 0.2 μm microporous membrane and stored at −80°C.

### 2.4 Screening of effective chemical components and analysis of the absorbed constituents

The ECP extract was appropriately diluted to a density of 0.28 g/mL, followed by mixing with an equal volume of methanol, 5‐min whirling and 20‐min centrifugal at 14,000 r/min. All frozen serum samples were thawed and equilibrated at 4°C prior to analysis. To detect the composition of the sample, 500 μL serum was combined with 1500 μL mixed solution (methanol: acetonitrile = 1:1) and vibrated for 2–3 min. After 20‐min vibration at 14,000 r/min, the mixture was then frozen dry. Finally, the re‐dissolution of freeze‐dried powder was made in 200 μL of methanol, followed by 20‐min subsequent centrifugal at 14,000 r/min. The supernatant was gathered for analytical experiments. The quantitative analysis was made by the UPLC‐QTRAP‐MS/MS system (Ultra performance liquid chromatography‐quadrupole‐time of ion trap mass spectrometer). Chromatographic condition was as follows: ACQUITY UPLC HSS T3 column (100 × 2.1 mm, 1.8 μm); mobile phases were set as A (HCOOH: CH_3_CN = 0.1: 100, v/v) and B (HCOOH: H_2_O = 0.1: 100, v/v): 0 ~ 0.5 min, 99% ~ 70%B; 0.5 ~ 5 min, 70% ~ 59%B; 5 ~ 5.5 min, 59% ~ 1%B; 5.5 ~ 8 min, 1%B; 8 ~ 8.1 min, 1% ~99%B; 8.1 ~ 10 min, 99%B; the flow rate was 0.3 mL/min; the injection tray temperature was 8°C; the column temperature was 40°C; the injection volume was 4 μL. MS condition was as follows: Ionization mode: electric spray ion source (ESI); Multi‐reaction monitoring ion scanning mode (MRM) detection; Ion Source Gas1 (Gas1): 55; Ion Source Gas2 (Gas2): 55; Curtain gas (CUR): 35; Source temperature: 500°C; IonSapary Voltage Floating (ISVF): −4500 V (positive/negative ion mode); The secondary mass spectrometry was obtained by MRM, and the MRM parameters are displayed in Table S1. The qualitative analysis was made by the UPLC‐QTOF‐MS/MS (Ultra performance liquid chromatography‐quadrupole‐time of flight‐mass spectrometer) system based on our previous experiment.^19^ Meanwhile, the method validation was performed according to some related studies.^21^

### 2.5 Network pharmacology approach

The mol2 files for Curculigoside, Baohuoside I, Epimedin A/B, Icariin and Orcinol glucoside were uploaded to PharmaMapper for predicting the relevant targets of bone repair. The keyword ‘bone healing’ was selected in the GeneCards database (https://www.genecards.org), OMIM database (http://www.omim.org) and the Drugbank database (https://www.drugbank.ca) to find out the relevant targets. The ‘VennDiagram’ package in R software was employed to intersect the hub genes between the absorbed constituents and bone repair. Moreover, the biological mechanisms of hub genes were offered by the Kyoto Encyclopedia of Genes and Genomes (KEGG) (https://www.kegg.jp/kegg/rest/keggapi.html) and the gene GO annotation in the R package ‘org.Hs.eg.db’ (version 3.1.0). All these analyses were made with the ‘clusterProfiler’ package in R software.

### 2.6 Cell culture

The MC3T3‐E1 cell line, derived from mouse calvaria pre‐osteoblasts, was adopted as a model of osteoblasts according to previous report^22^ (collectively referred to as osteoblasts). All osteoblasts during the experiment were cultured in α‐MEM medium (Genomcell bio; China) containing 10% fetal bovine serum. Osteoblasts were grown in a humidified incubator (95% air, 5% CO_2_ at 37°C). Fresh medium was utilized and changed every 2–3 days. Upon reaching 80% confluency, the cells were digested, counted and prepared into cell suspensions in different culture vessels for additional studies.

### 2.7 CCK‐8 assay

The cytotoxicity of LPS and the therapeutic effect of ECP‐BS/ECP‐CS on osteoblasts was assessed by the CCK‐8 assay. Osteoblasts were put in a 96‐well plate at a concentration of 5 × 10^4^ cells/mL. Subsequently, the treatment of osteoblasts with (0–100 μg/mL) LPS or (0–20%) ECP‐BS/ECP‐CS was made at 24 h and 48 h, respectively. Then, osteoblasts were exposed to CCK‐8 solution and analysed with a microplate reader at λ = 450 nm (Thermo Fisher Scientific Inc, USA).

### 2.8 Cell transfection and grouping

The following study utilized synthesized vectors, including empty vector (pcDNA3.1), siMALAT1 (pcDNA3.1‐MALAT1), Negative Control (NC), miR‐34a‐5p mimic, inhibitor NC, miR‐34a‐5p inhibitor and siSMAD2, all provided by GenePharma (Shanghai, China). Cell seeding was made in a six‐well plate with the concentration of 5 × 10^5^ cells/mL. When reached 60%, cells were cultured with opti‐MEM (Grand Island Biological Company, USA) containing transfection working solution (500 μL/well) for 6 h. After post‐transfection, cells were refreshed with fresh medium for 24 or 48 h at 37°C with 5% CO_2_. The sequence of plasmids adopted in the manuscript was given in Table S2.

### 2.9 Quantitative real‐time PCR (qRT‐PCR) assay

The extraction of total RNA was made from osteoblasts by SPARKeasy Cell RNA Rapid Extraction Kit (Invitrogen, USA) and subsequently transcribed into cDNA based on SPARKscriptIIRT Plus Kit (With gDNA Eraser) or SPARKScript II miRNA 1st strand cDNA synthesis Kit (By stem‐loop) (Hangzhou Diante Biotechnology Co Ltd, China). The expressions of MALAT1, miR‐34a‐5p and mRNAs of SMAD2, ALP, RUNX2 were tested by the 2 × SYBR Green qPCR Mix (With ROX) or 2 × miRNA SYBR Green qPCR Mix (By stem‐loop) (Hangzhou Diante Biotechnology Co Ltd, China). In each of the samples, the reference control gene utilized was β‐actin, while U6 was implemented as the internal control to account for miR‐34a‐5p. Quantitative real‐time PCR was made with the Biosystems 7500 Real‐Time PCR system. The 2^−ΔΔCT^ approach was adopted for calculating the relative gene expression data. Table S3 lists the primers adopted in this research.

### 2.10 Western blot assay

The extraction and lysis of total proteins were made through the use of RIPA lysis buffer and were subsequently quantified. Subsequently, the transfer of proteins underwent separation via SDS‐PAGE, was made onto polyvinylidene difluoride membranes (PVDF) (Millipore, Bedford, MA, USA), next to 1‐h blocking with 5% BSA at room temperature. Meanwhile, the incubation of PVDFs was made successively with primary antibodies anti‐SMAD2 (1:500, bs‐0718R, Bioss, USA), anti‐ALP (1:500, bsm‐52252R, Bioss, USA), anti‐RUNX2 (1:500, db20178, diagbio, China), anti‐collagen type I (1:500, PB0981, Boster, China) and anti‐GAPDH (1:1000, A00227‐1, Boster, China) at 4°C overnight. Then, the PVDFs were subjected to an hour‐long incubation with HRP‐labelled secondary antibodies (1:2000, BA1054, Boster, China) for 1 h at room temperature. The protein levels were examined through ChemiDocTM MP Imaging system (BioRad Co., Ltd., CA, USA), followed by analysis with the image J software.

### 2.11 Cell differentiation and mineralization assay

The ALP staining was utilized to assess cell differentiation capacity. Osteoblasts were cultured in six‐well plates and treated accordingly. After treatment for 24 h, pre‐cooled PBS was adopted to wash cells for three times before fixation with 4% paraformaldehyde at 37°C for 10 min and, next to staining by the ALP staining kit (20210520; KGI Biotechnology, China).

The Alizarin Red S staining was made to evaluate cell mineralization capability. To induce calcification, osteoblasts were cultured in induction medium containing ascorbic acid (50 μg/L, A171447, Aladdin, China), β‐Sodium glycerophosphate (10 mmol/L, BCCD6874, Sigma‐Aldrich, USA) and dexamethasone (1 × 10^−8^ mmol/L, B1704084, Aladdin, China) for 21 days. The medium was refreshed at 48‐h intervals during induction. After successful calcification, cells were fixed by the same method mentioned above and then stained with 0.1% Alizarin Red S solution (130‐22‐3, Aladdin, China) for 1 h at 37°C. The calcium nodules appeared as orange or red nodules were then discovered under a microscope (Nikon Eclipse Ti, 531,608).

### 2.12 Dual‐luciferase reporter assay

The Starbase (http://starbase.sysu.edu.cn/) was employed to predict the binding sites of LncRNA MALAT1 and miRNA‐34a‐5p. The mutated type sequences (TTGGACTGTTATCATATATTAAATGTATCGGTTTCGTGACGGACCTGCTCTGTGCACTTGAAAGGATCCC) and wild‐type sequences (TTGGACTGTTATCATATATTAAATGTATGCGTATGCACTGCCACCTGCTCTGTGCACTTGAAAGGATCCC) in the predicted binding sites were designed (WT‐MALAT1 and MUT‐MALAT1) and cloned into luciferase expression vector (GP‐miRGLO) by GenePharm Pharmaceutical Technology Co., Ltd. (Shanghai, China).

For the luciferase assay, HEK 293 cells were plated in 96‐well plates and cultured for 24 h. Subsequently, cells were co‐transfected with 50 nM of either Negative Control or miRNA‐34a‐5p mimic, along with the dual‐luciferase vectors containing either WT‐MALAT1 or MUT‐MALAT1, using the LipoHigh transfection reagent (GenePharm Pharmaceutical Technology Co., Ltd. Shanghai, China) for 5 h. After transfection, the cells were refreshed with α‐MEM medium containing 10% fetal bovine serum and incubated at 37°C with 5% CO_2_ for 24 h.

Following the protocols outlined in the dual‐luciferase reporter assay kit (Yeasen Biotechnology Co., Ltd., Shanghai, China), the cells were lysed using Passive Lysis Buffer and added to the Firefly luciferase reaction solution for 30 min. The activity of Firefly luciferase (F) was then measured. Subsequently, the Renilla luciferase reaction solution was added, and the mixture was thoroughly mixed using a vibrator to detect the activity of Renilla luciferase (R) within 30 min. Finally, the association between miRNA‐34a‐5p and LncRNA MALAT1 was quantitatively evaluated by calculating the ratio of Renilla luciferase activity to Firefly luciferase activity.

### 2.13 Statistical analysis

In this study, the results of our analysis were displayed as the mean ± SD and analysed by GraphPad Prism 6.0 (GraphPad Software, San Diego, CA, USA). Variations between multiple groups were decided by one‐way ANOVA followed. Statistical significance was considered at *p* < 0.05 and *p* < 0.01.

## 3 RESULTS

### 3.1 ECP reversed the inhibition of bone formation caused by IBDs

**FIGURE 1:**
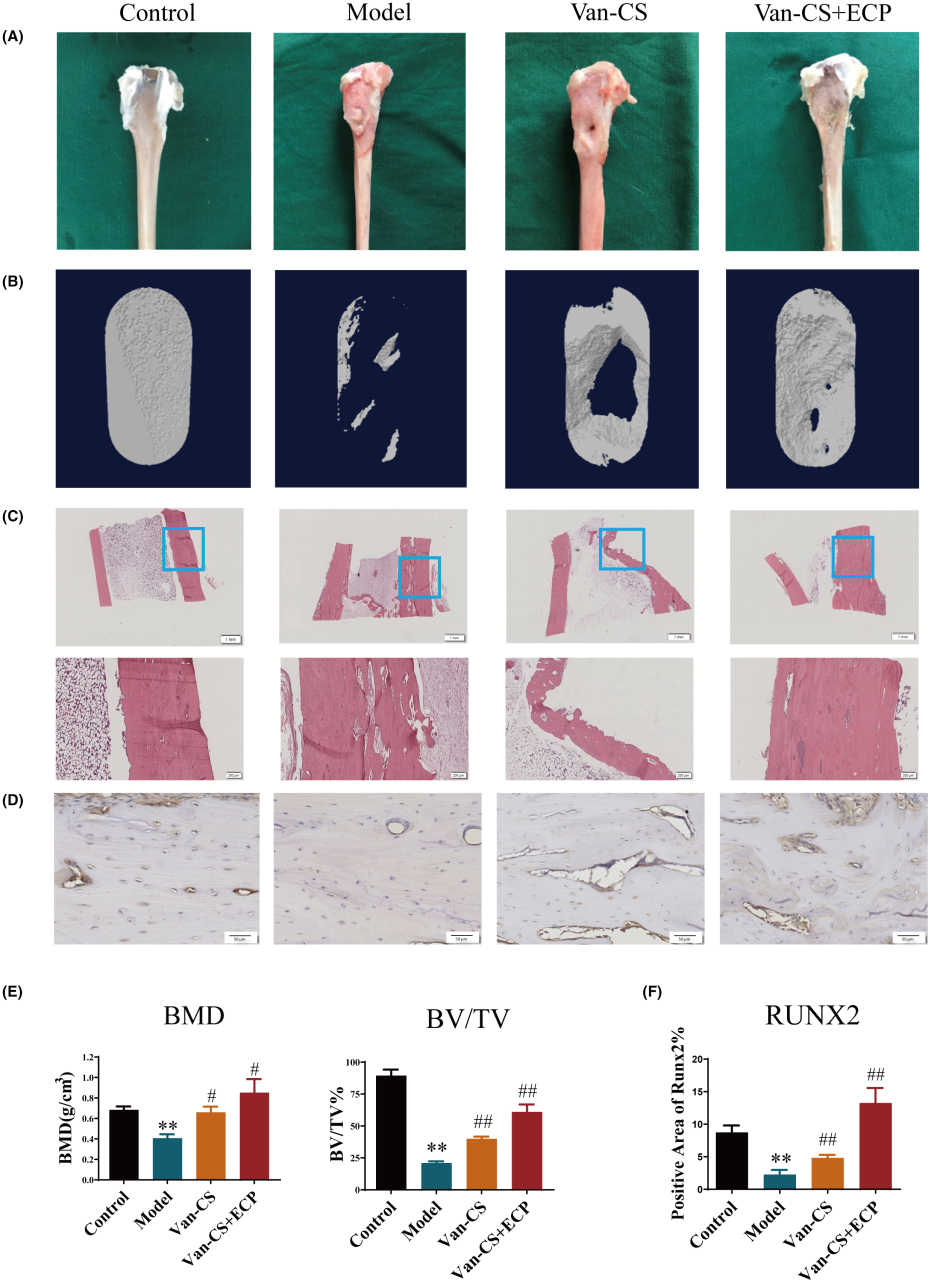
The bone repair effect of ECP in IBDs. (A) The appearance observation of tibia specimens in each group. (B) The micro‐CT scanning images of lesion sites in each group. (C) The HE staining images of tibia specimens (10×, scale bar = 1 mm, 40×, scale bar = 200 μm). (D) The IHC staining images of BMP‐2 in each group (200×, scale bar = 50 μm). (E) The BV/TV and BMD indexes (F) The protein degrees of RUNX2 in bone tissue. (*p < 0.05, **p < 0.01, vs. Control group; ^#^ *p* < 0.05, ^##^ *p* < 0.01, vs. Model group; *n* = 3).

To explore the bone repair effect of ECP, we analysed bone tissue samples from each group. The results showed that there was obvious bone loss in Model group by comparing with Control group. In the Van‐CS group, a modest amount of fresh bone was present at the defect site, but the defect cavity was still visible. Nevertheless, the cortical bone structure of the Van‐CS + ECP group had become complete and continuous, and the defect cavity was reverted to its normal state essentially (Figure 1A,B). According to statistical analysis, Model group displayed greatly lower BV/TV and BMD than Control group (Figure 1E). Besides, Van‐CS + ECP group displayed higher BV/TV and BMD than Model and Van‐CS groups significantly (Figure 1E). These findings indicated that ECP could significantly increase bone repair in IBDs.

According to the HE staining results, a lot of inflammatory cells were found in the bone tissue of the Model group by comparing with Control group (Figure 1C). The inflammatory state of the Van‐CS group was mitigated, while the bone defect was aggravated. Interestingly, the bone cortex of Van‐CS + ECP group was substantially improved, and the inflammatory cells between the bone tissues were greatly decreased (as shown in Figure 1C). On the other hand, IHC staining results indicated that the expressions of RUNX2, identified as the early osteoblastic marker, in Model group were greatly decreased by comparing with Control group. Moreover, Van‐CS and Van‐CS + ECP groups showed greatly higher the protein expressions of RUNX2 than Model group. It was worth mentioning that the positive expressions of RUNX2 in the Van‐CS + ECP group were higher than that in Van‐CS group (Figure 1D,F). These outcomes hinted that ECP could significantly grow the expressions of RUNX2 to facilitate bone repair in IBDs.

### 3.2 Chemical constituents' characterization in ECP extract and Quantification of 6 components in ECP‐CS

In order to analyse the chemical constituents' characterization in ECP extract and ECP‐CS, the UPLC‐QTOF‐MS/MS methods were employed. The results showed that 34 constituents were identified in the ECP extract (Tables S4–S6, Figure S1), and 6 constituents (Curculigoside, Baohuoside I, Epimedin A/B, Icariin and Orcinol glucoside) (Table S7, Figure S2) were verified to absorb into the serum. Furthermore, 6 absorbed constituents in ECP‐CS were quantitative analysed by using UPLC‐QTRAP‐MS/MS methods. First of all, as shown in Table S8 and Figure S3, the selectivity, accuracy, precision, stability, extraction recovery and matrix effect of UPLC‐QTRAP‐MS/MS approach were met the requirements, except the low‐concentration icariin couldn't be detected. It indicated that the UPLC‐QTRAP‐MS/MS approach was feasible to detect the concentrations of the 6 components. Finally, the results showed that the concentrations of Baohuoside I, Icariin, Epimedin B, Orcinol glucoside, Curculigoside and Epimedin A in serum were 2.99 ng/mL, 0.94 ng/mL, 18.14 ng/mL, 1581.00 ng/mL, 4.52 ng/mL and 3.58 ng/mL, respectively (as shown in Table 1).

**TABLE 1:** Regression, equation and concentration for the six absorbed constituents.

Components	Linear regression equation	*r* ^2^	Range (ng/mL)	Concentration (ng/mL)
Baohuoside I	*y* = 15646.6662 *x* + 3728.2950	0.9995	0.6300–10.0000	2.9933 ± 0.1457
Icariin	*y* = 2843.6066 *x* + 42256.5000	0.9981	4.3800–140.0000	0.9367 ± 0.9441
Epimedin B	*y* = 1715.1807 *x* + 345.1314	0.9981	0.1600–160.0000	18.1367 ± 1.1736
Orcinol glucoside	*y* = 4093.1744 *x* + 19663.7416	0.9985	7.8100–4000.0000	1581.0000 ± 27.0554
Curculigoside	*y* = 5815.0749 *x* + 283.7228	0.9990	0.1600–10.0000	4.5167 ± 0.1419
Epimedin A	*y* = 1485.7528 *x* + 63.0124	0.9988	0.1600–10.0000	3.5800 ± 0.3579

### 3.3 Network pharmacology predicted the therapeutic targets of the absorbed constituents in the process of inflammatory and bone repair

**FIGURE 2:**
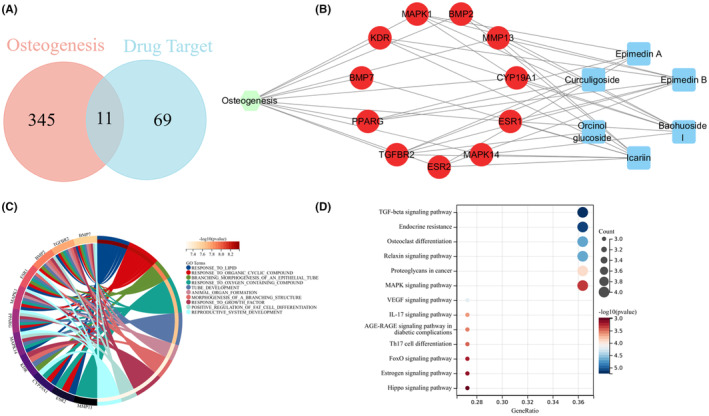
Network pharmacology‐based prediction of the six absorbed constituents. (A) Intersection of the six absorbed constituents' targets with osteogenesis‐related genes involved in treating bone repair by Venn diagram. (B) Network diagram of osteogenic‐core targeted of the 6 absorbed constituents. (C) A circle diagram showed an analysis of the biological processes involved in the core targets of the six absorbed constituents by GO. (D) Analysis of the pathways involved in the core targets of the six absorbed constituents by the KEGG and bubble diagrams.

Based on the results of GeneCards and OMIM databases, there were 356 genes related to bone repair, and 80 genes were related to 6 absorbed constituents (Curculigoside, Baohuoside I, Epimedin A/B, Icariin and Orcinol glucoside). Then, 11 hub genes were sought out by intersecting bone repair related genes and 6 components target genes using the ‘VennDiagram’ package in R software (Figure 2A,B). The main hub targets genes were MMP13, BMP7, MAPK14, BMP2, TGFBR2, KDR, PPARG, ESR1, MAPK1, ESR2 and CYP19A1. Meanwhile, the GO annotation and KEGG enrichment analysis were adopted for exploring the biological function of these hub targets. The Network pharmacology prediction results showed that 64 biological process (BP) and 10 signalling pathways were related with bone repair and inflammatory, such as osteogenic TGF‐β signalling pathway, Hippo signalling pathway, angiogenesis related VEGF signalling pathway, MAPK and IL‐17 signalling pathway (Figure 2C,D). Most importantly, the 6 components in the ECP have been proved to be an effective treatment for bone repair through intervening osteogenesis, angiogenesis or anti‐inflammatory function. Above all, according to the preliminary experiments and literature searches, TGF‐β/SMADs signalling pathway was selected for further research.

### 3.4 LncRNA MALAT1 regulated genes involved in the differentiation and mineralization of osteoblasts

**FIGURE 3:**
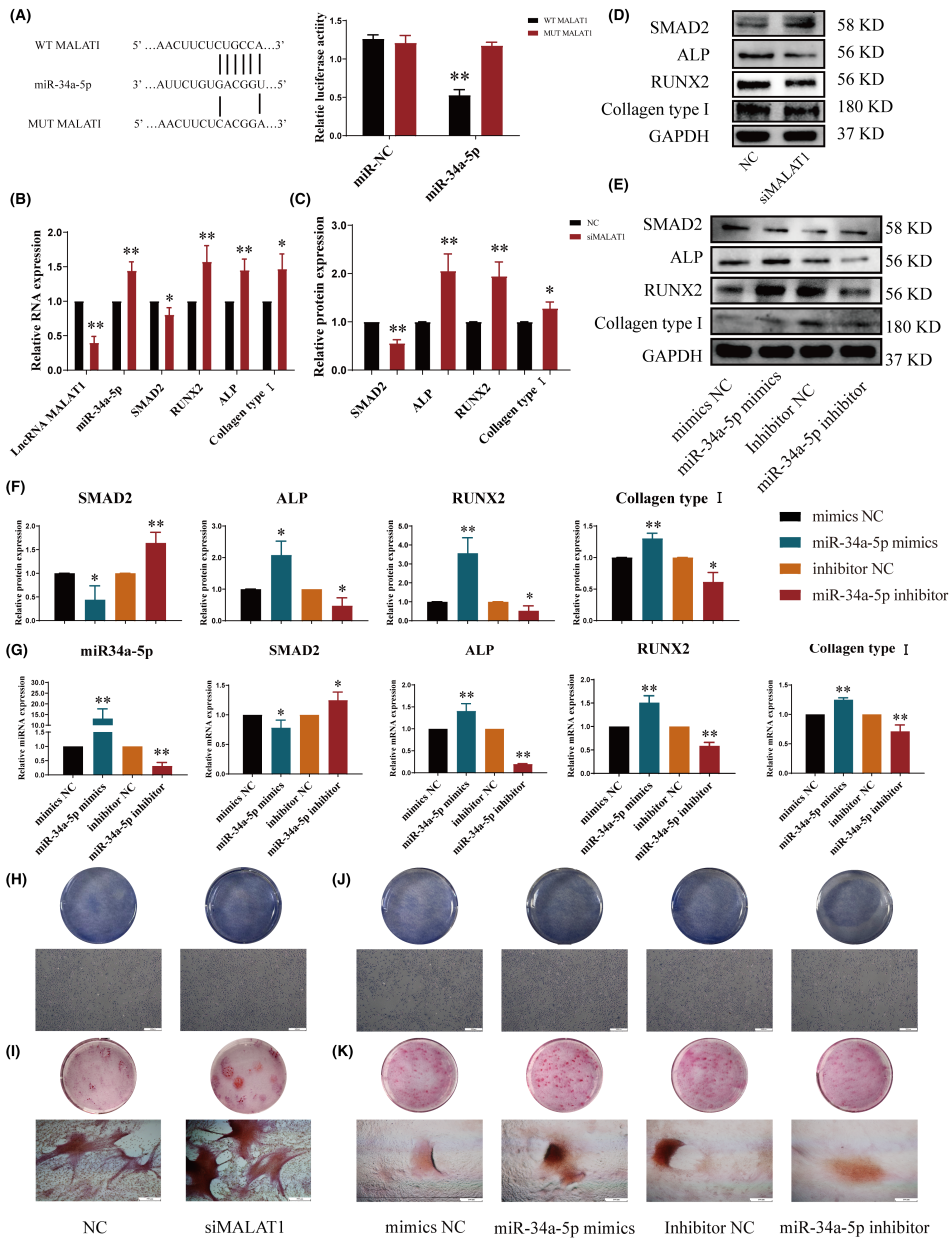
siMALAT1 and miR‐34a‐5p mimics drove the differentiation and mineralization of osteoblasts. (A) StarBase predicted the binding site between LncRNA MALAT1 and miR‐34a‐5p; Diagram of putative miR‐34a‐5p binding sequence in LncRNA MALAT1 3′UTR and its mutant in luciferase reporter assay. (B, G) qRT‐PCR was used to test the gene expression of LncRNA MALAT1, miR‐34a‐5p, SMAD2, ALP, RUNX2 and Collagen type І in osteoblasts. (C–F) Western Blot was used to test the protein expression of SMAD2, ALP, RUNX2 and Collagen type І. (H, J) The differentiation abilities of osteoblasts were observed by ALP staining (40×, scale bar = 500 μm). (I, K) The mineralization abilities of osteoblasts were observed by Alizarin red (40×, scale bar = 500 μm). (*p < 0.05, **p < 0.01, vs. NC or mimics NC; *n* = 3).

According to the TargetScan (http://www.targetscan.org/vert_80/) prediction, miR‐34a‐5p had LncRNA MALAT1 binding places in its 3′UTR. In order to investigate whether LncRNA MALAT1 directly targets miR‐34a‐5p, we constructed luciferase reporter genes with LncRNA MALAT1, including wild‐type (WT) or mutant (MUT). The findings of dual‐luciferase reporter assay unveiled that upregulation of miRNA‐34a‐5p greatly impeded the luciferase reporter activity of the vector with the WT LncRNA MALAT1 3′UTR (Figure 3A). In summary, LncRNA MALAT1 could directly regulate the expression of miR‐34a‐5p.

To dig the biological role of LncRNA MALAT1 in osteoblasts, low‐expression plasmid siMALAT1 was transferred into osteoblasts. The qRT‐PCR outcomes displayed that low‐expression of LncRNA MALAT1 could sensibly increase the gene expression of miR‐34a‐5p, while obstruct the gene expression of SMAD2. Meanwhile, the gene expressions of ALP, RUNX2 and Collagen type І, markers of differentiation and mineralization in osteoblasts, were significantly increased as well (Figure 3B). As shown in Figure 3C,D, the results of Western Blot were consistent with that of qRT‐PCR. The protein expression of SMAD2 was decreased, while the protein expressions of ALP, RUNX2 and Collagen type І were grown. Meanwhile, the differentiation and mineralization of osteoblasts were observed macroscopically by Alizarin Red S staining and ALP staining. Compared with NC group, siMALAT1 could dramatically increase the number of osteoblasts and the degree of mineralized nodules (as shown in Figure 3H,I).

Based on the experimental results, it was evident that knockdown of lncRNA MALAT1 significantly suppressed the expression of osteoblasts' signature genes, thereby inhibiting the differentiation and mineralization abilities. Additionally, LncRNA MALAT1 could directly regulate the expression of miR‐34a‐5p and impacted the expression of SMAD2. Thus, the above outcomes suggested that LncRNA MALAT1 could regulate osteoblastic differentiation and mineralization by mediating miR‐34a‐5p.

### 3.5 MiR‐34a‐5p regulated genes involved in the propagation and mineralization of osteoblasts

For the investigation into the function of miR‐34a‐5p, we constructed miR‐34a‐5p mimics plasmid and inhibitor plasmids. According to the qRT‐PCR results, overexpression of miR‐34a‐5p could greatly reduce the gene expression of SMAD2, while increase the gene expressions of ALP, RUNX2 and Collagen type І. The miR‐34a‐5p inhibitor had exactly the opposite effects on osteoblasts (Figure 3G). Western Blot outcomes also demonstrated that miR‐34a‐5p inhibitor drove the protein expression of SMAD2, but inhibited the protein expressions of ALP, RUNX2 and Collagen type І (Figure 3E,F). Similarly, as shown in Alizarin Red S staining and ALP staining results, miR‐34a‐5p mimics drove the abilities of propagation and mineralization in osteoblasts, compared with NC group (Figure 3J,K).

Our findings demonstrated that upregulating the expression of miR‐34a‐5p could enhance the expression of osteoblasts' genes, which associated with differentiation and mineralization, thereby promoting the osteogenic differentiation and mineralization process. Besides, SMAD2 was downregulated as well. If miR‐34a‐5p was inhibited, the opposite would be true. Furthermore, our previous experiments have validated the targeted binding between miRNA and SMAD2. Collectively, our results indicated that miR‐34a‐5p might drive the propagation and differentiation of osteoblasts by mediating the expression level of SMAD2.

### 3.6 SMAD2 regulated genes involved in the propagation and mineralization of osteoblasts

**FIGURE 4:**
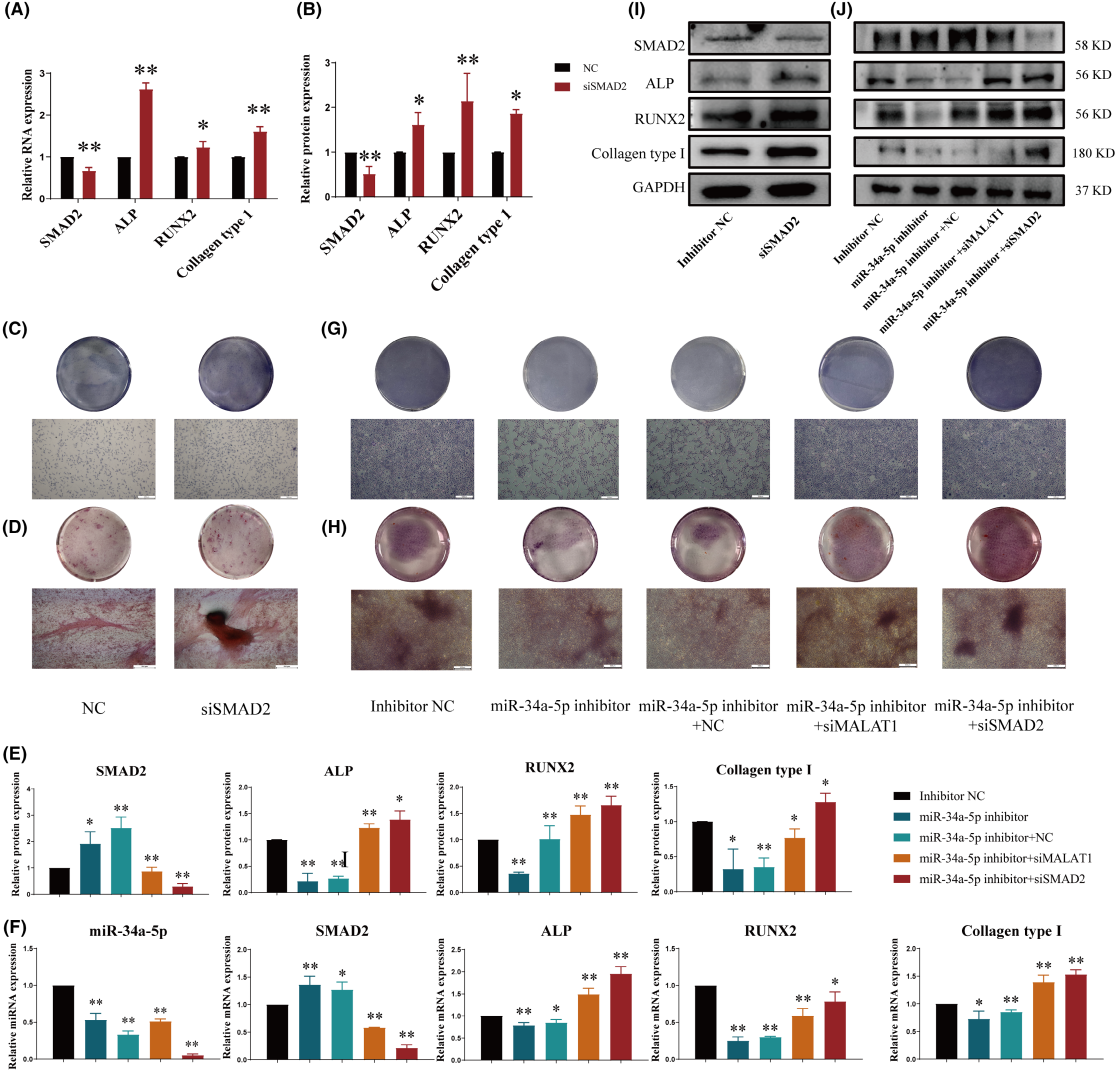
Verification of LncRNA MALAT1/miR‐34a‐5p/SMAD2 signalling pathway. (A, F) qRT‐PCR was adopted to detect the gene expressions of miR‐34a‐5p, SMAD2, ALP, RUNX2 and Collagen type І in osteoblasts. (B, E, I, J) Western Blot was adopted for testing the protein expressions of SMAD2, ALP, RUNX2 and Collagen type І. (C, G) The differentiation abilities of osteoblasts were discovered by ALP staining (40×, scale bar = 500 μm). (D, H) The mineralization abilities of osteoblasts were observed by Alizarin red staining (40×, scale bar = 500 μm). (*p < 0.05, **p < 0.01, vs. NC or inhibitor NC; *n* = 3).

In order to determine the bioactive function of SMAD2, the siSMAD2 plasmid was constructed. The gene expressions of ALP, RUNX2 and Collagen type І were significantly increased (Figure 4A), and the corresponding protein expressions were remarkably raised (Figure 4B,I) when siSMAD2 was transferred into osteoblasts. Moreover, siSMAD2 dominated in promoting the propagation and mineralization of osteoblasts compared with NC group, as confirmed by Alizarin Red S staining and ALP staining (Figure 4C,D).

These results indicated that Low‐expression SMAD2 could directly stimulate the expression levels of osteoblasts' signature genes, related to osteoblast proliferation and mineralization capacity, consequently impacting osteoblasts' biological functions. These outcomes strongly showed that the knockout of SMAD2 promoted the differentiation and differentiation of osteoblasts.

### 3.7 Elimination of LncRNA MALAT1 or SMAD2 counteracted the inhibitory role of miR‐34a‐5p inhibitor in propagation and mineralization of osteoblasts

In order to verify the feasibility of the LncRNA MALAT1/miR‐34a‐5p/SMAD2 signalling pathway, miR‐34a‐5p inhibiting agent was selected to verify whether siMALAT1 and siSMAD2 could reverse its activities. As shown in Figure 4F, the gene expressions of SMAD2, ALP, RUNX2 and Collagen type І in miR‐34a‐5p Inhibitor + siMALAT1 group and miR‐34a‐5p Inhibitor + siSMAD2 group were markedly higher than those in miR‐34a‐5p inhibitor group. Meanwhile, the protein expressions of SMAD2, ALP, RUNX2 and Collagen type І were dramatically elevated compared with miR‐34a‐5p inhibitor group as well (Figure 4E,J). The outcomes of Alizarin Red S staining and ALP staining displayed that miR‐34a‐5p Inhibitor + siMALAT1 group and miR‐34a‐5p Inhibitor + siSMAD2 group could increase the number of osteoblasts and mineralized nodules, which reverse the damage caused by miR‐34a‐5p inhibitor (Figure 4G,H).

Interestingly, we found that we could partially rescue the inhibitory effect of miR‐34a‐5p on osteoblasts by inhibiting the expression of LncRNA MALAT1, which was the upstream factor regulating miR‐34a‐5p. However, if we specifically targeted and suppressed the downstream target of miR‐34a‐5p—SMAD2, it would be feasible to reinstate the diminished osteogenic differentiation and mineralization capacities, which are otherwise compromised by the suppressive effects of miR‐34a‐5p, to physiological standards. The above results deduced that LncRNA MALAT1 /miR‐34a‐5p /SMAD2 signalling pathway regulated the mineralization and differentiation of osteoblasts.

### 3.8 LPS induced the proliferation and mineralization of osteoblasts

**FIGURE 5:**
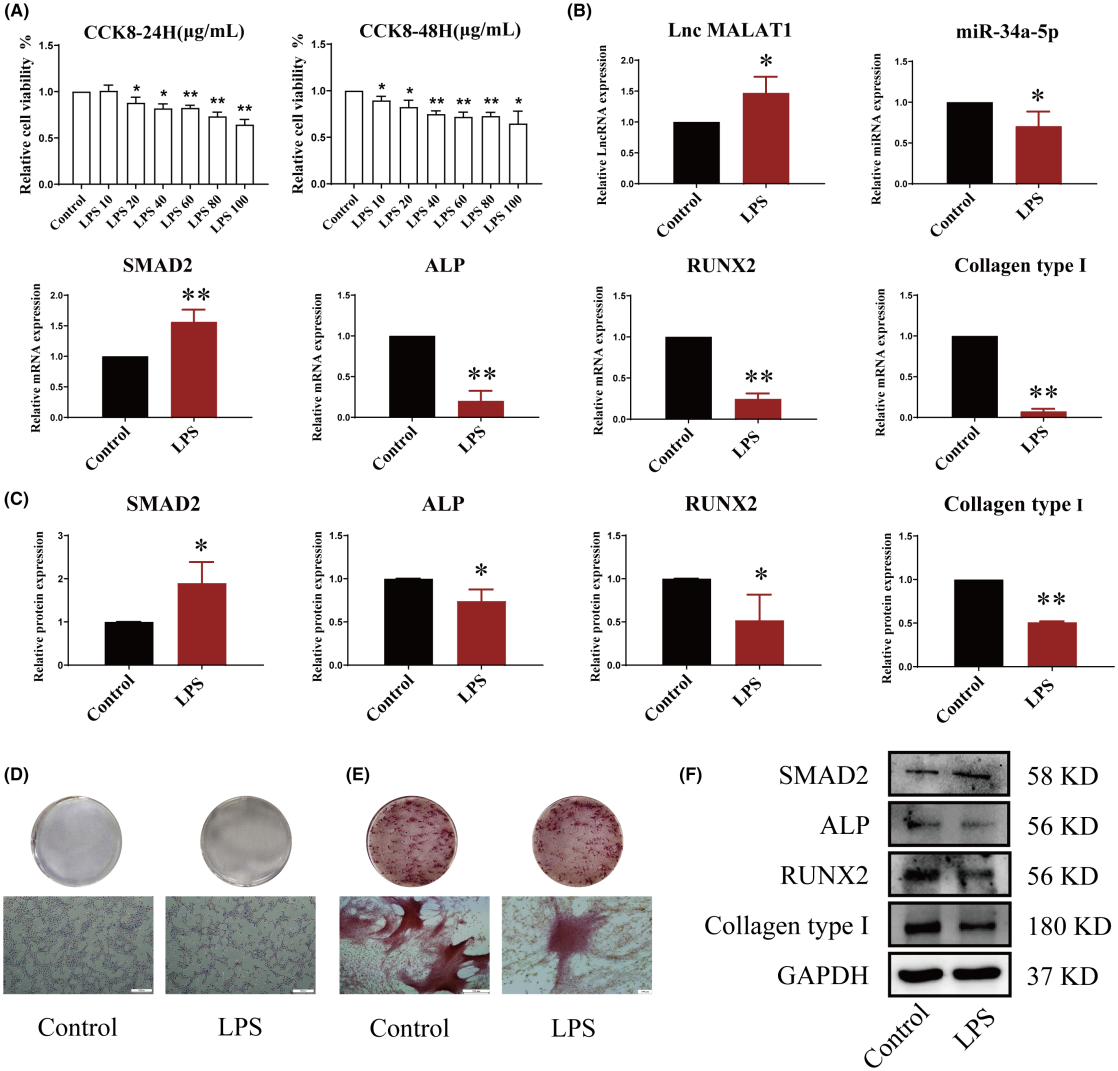
LPS inhibited the differentiation and mineralization of osteoblasts. (A) Effect of LPS on differentiation of osteoblasts was detected by CCK‐8. (B) qRT‐PCR was used to detect the gene expressions of LncRNA MALAT1, miR‐34a‐5p, SMAD2, ALP, RUNX2 and Collagen type І in osteoblasts. (C, F) Western Blot was employed for detecting the protein expressions of total SMAD2, ALP, RUNX2 and Collagen type І. (D) The differentiation abilities of osteoblasts were observed by ALP staining (40×, scale bar = 500 μm). (E) The mineralization abilities of osteoblasts were observed by Alizarin red (40×, scale bar = 500 μm). (*p < 0.05, **p < 0.01, vs. Control, *n* = 3).

To assess the cytotoxicity of LPS, osteoblasts were incubated with 0, 10, 20, 40, 60, 80 or 100 μg/mL LPS for 24 h and 48 h, and the cell survival rates were measured by a microplate reader. As shown in Figure 5A, osteoblasts' proliferation abilities were greatly reduced by LPS in a dose‐dependent way and reached the plateau stage at 100 μg/mL gradually. The cell survival rate of 100 μg/mL group was decreased from 100.00% to 59.86% and 65.8% at 24 h and 48 h separately (Figure 5A). Therefore, the 100 μg/mL was selected as the LPS inducing concentration.

Other experiments were made to dig the role of LPS in the differentiation and mineralization of osteoblasts. The outcomes of qRT‐PCR displayed that LPS could greatly increase the gene expressions of LncRNA MALAT1 and SMAD2, while reduce the gene expressions of miR‐34a‐5p, ALP, RUNX2 and Collagen type І (Figure 5B). Western blot outcomes suggested that LPS grew the protein expression of SMAD2 and declined the protein expressions of ALP, RUNX2 and Collagen type І substantially (Figure 5C,F). The outcomes of Alizarin Red S staining and ALP staining displayed that LPS memorably reduced the number of osteoblasts and the size and degree of mineralized nodules by comparing with the control group (Figure 5D,E). The above outcomes implied that LPS regulated the differentiation and mineralization of osteoblasts by mediating the LncRNA MALAT1 /miR‐34a‐5p/ SMAD2 signalling pathway.

### 3.9 LPS induced the differentiation and mineralization of osteoblasts through LncRNA MALAT1/miR‐34a‐5p/SMAD2 axis

**FIGURE 6:**
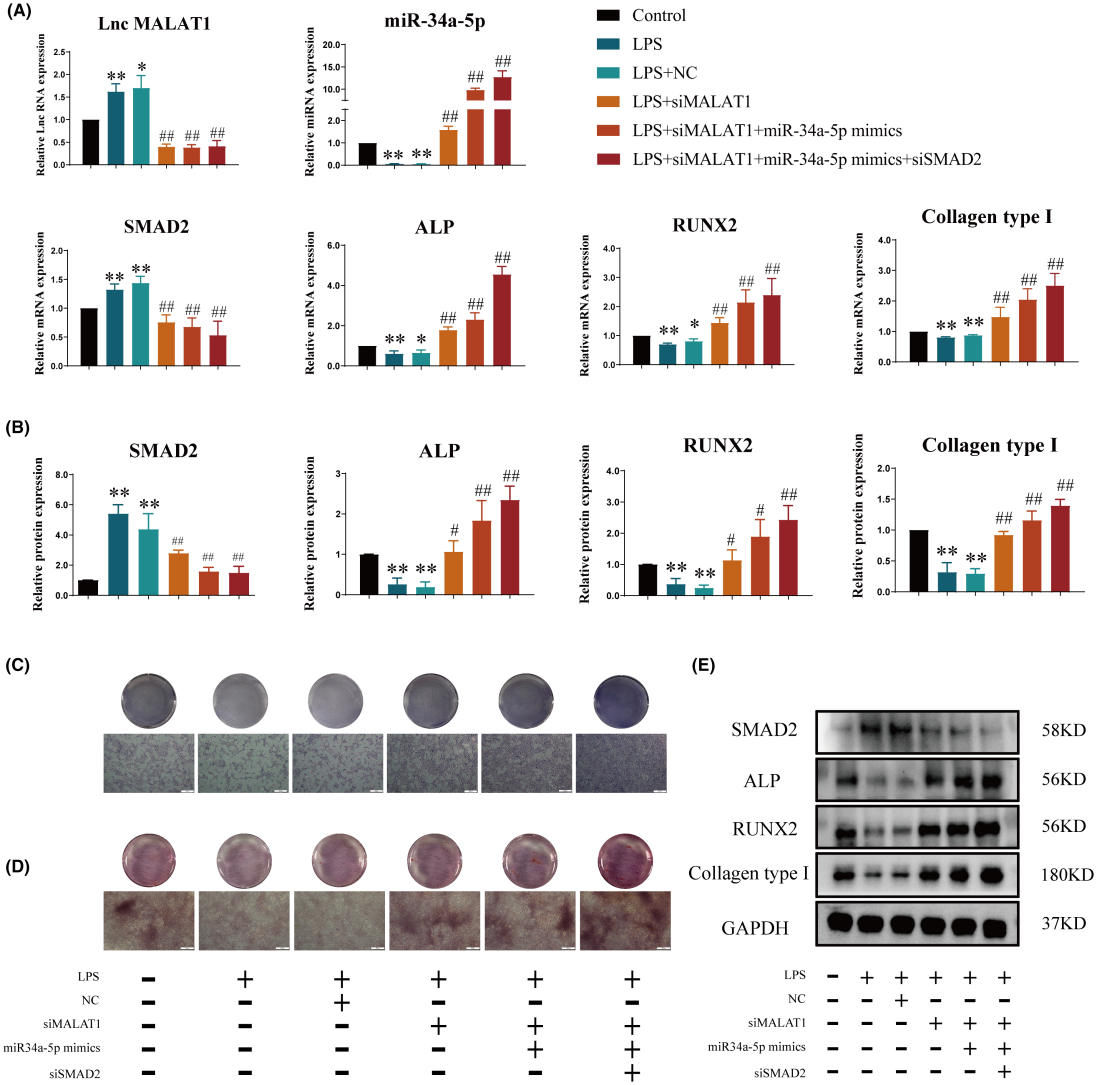
Verification of that LPS mediated LncRNA MALAT1/miR‐34a‐5p/SMAD2 signalling pathway on osteoblasts. (A) qRT‐PCR was employed for detecting the gene expression of miR‐34a‐5p, SMAD2, ALP, RUNX2 and Collagen type І in osteoblasts. (B, E) Western Blot was employed for detecting the protein expression of total SMAD2, ALP, RUNX2 and Collagen type І. (C) The differentiation abilities of osteoblasts were observed by ALP staining (40×, scale bar = 500 μm). (D) The mineralization abilities of osteoblasts were observed by Alizarin red (40×, scale bar = 500 μm). (*p < 0.05, **p < 0.01, vs. Control; ^#^ *p* < 0.05, ^##^ *p* < 0.01, vs. LPS; *n* = 3).

In order to prove whether the LncRNA MALAT1/miR‐34a‐5p/SMAD2 signal axis exerted an effect on LPS‐cultured osteoblasts a set of molecular experiments were made. The results of qRT‐PCR showed that when siMALAT1 was transferred into osteoblasts, the gene expressions of LncRNA MALAT1 and SMAD2 in LPS + siMALAT1 group were significantly reduced, while the gene expressions of miR‐34a‐5p, ALP, RUNX2 and Collagen type І were grown (Figure 6A), compared with LPS group. Meanwhile, LPS + siMALAT1 group could downregulate the protein expression of SMAD2 and upregulate the protein expressions of ALP, RUNX2 and Collagen type І (Figure 6B,E). The results of Alizarin Red S staining and ALP staining got essentially the same results. LPS + siMALAT1 group improved the number of cells in osteoblasts and the size and extent of mineralized nodules (Figure 6C,D). After that, simultaneously transferring into siMALAT1 and miR‐34a‐5p mimics, the gene expressions of miR‐34a‐5p, SMAD2, ALP, RUNX2 and Collagen type І were grown and the gene expression of SMAD2 was declined memorably (Figure 6A). Meanwhile, the protein expressions of ALP, RUNX2 and Collagen type І were more improved, the protein expression of SMAD2 was further reduced (Figure 6B,E). Subsequently, it could have a better effect of the gene and protein expressions of SMAD2, ALP, RUNX2 and Collagen type І (Figure 6A,B,E), when siMALAT1, miR‐34a‐5p mimics and siSMAD2 worked together. The outcomes of Alizarin Red S staining and ALP staining showed that the differentiation and mineralization ability of osteoblasts in LPS + siMALAT1 + miR‐34a‐5p mimics group was more improved than those of LPS + siMALAT1 + miR‐34a‐5p mimics + siSMAD2 group (Figure 6C,D). These results insinuated that LPS mediated the differentiation and mineralization of osteoblasts through LncRNA MALAT1/miR‐34a‐5p/SMAD2 signal axis.

### 3.10 ECP reversed the inhibition of osteoblasts brought by LPS

**FIGURE 7:**
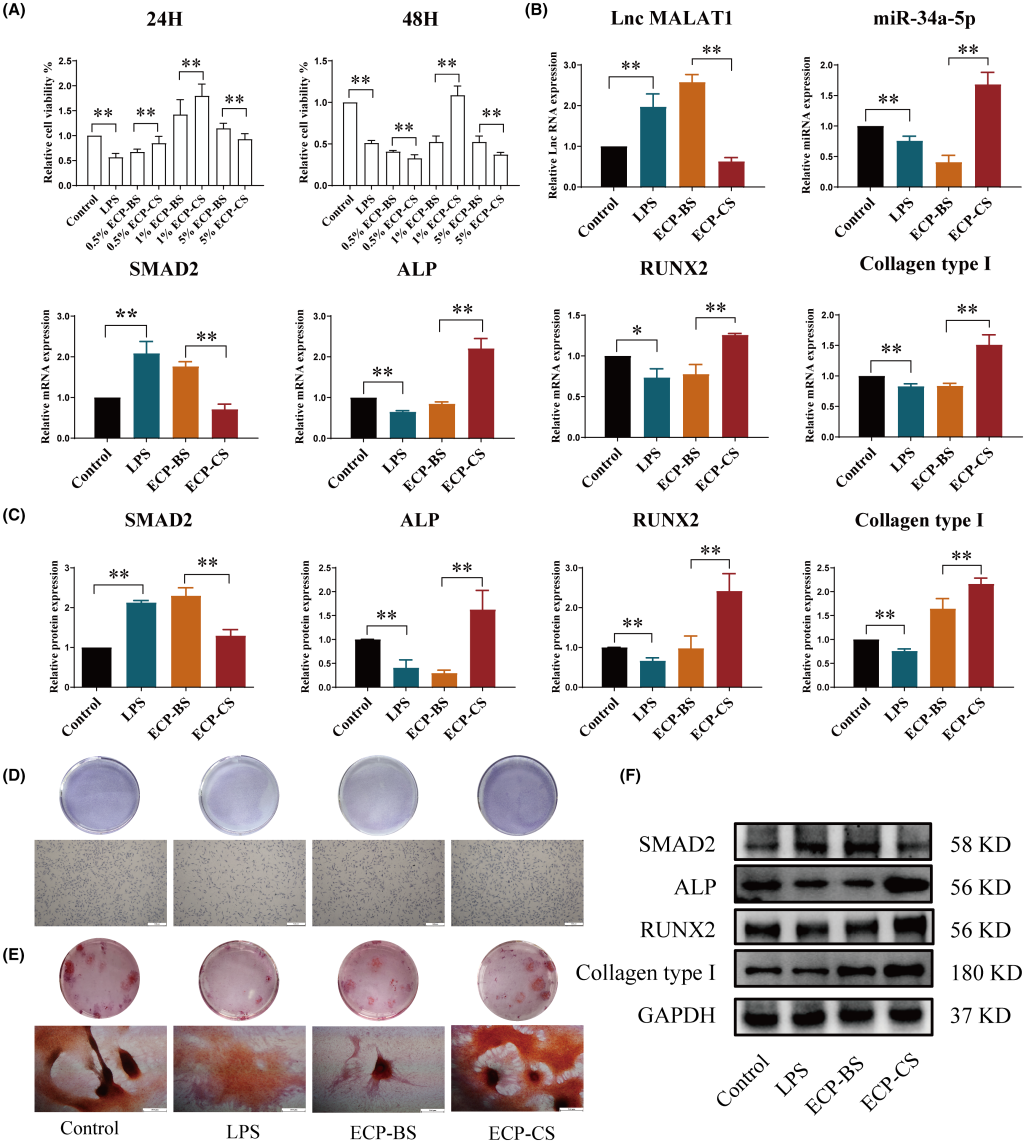
ECP‐CS reversed the inhibition of osteoblasts caused by LPS. (A) Effect of ECP‐CS on differentiation of osteoblasts was detected by CCK‐8. (B) qRT‐PCR was used to detect the gene expressions of LncRNA MALAT1, miR‐34a‐5p, SMAD2, ALP, RUNX2 and Collagen type І in osteoblasts. (C, F) Western Blot was used to test the protein expressions of SMAD2, ALP, RUNX2 and Collagen type І. (D) The differentiation abilities of osteoblasts were observed by ALP staining (40×, scale bar = 500 μm). (E) The mineralization abilities of osteoblasts were observed by Alizarin red (40×, scale bar = 500 μm). (*p < 0.05, **p < 0.01, vs. NC or ECP‐BS, *n* = 3).

As shown in Figure 7A, CCK8 assay revealed that the 1% ECP‐CS could markedly increase the cell viability of osteoblasts induced by LPS, comparing with LPS and ECP‐BS groups. The qRT‐PCR outcomes displayed that the gene expressions of LncRNA MALAT1 and SMAD2 were greatly decreased, while the gene expressions of miR‐34a‐5p, ALP, RUNX2 and Collagen type І were grown (Figure 7B). Meanwhile, the protein expression of SMAD2 was downregulated, and the protein expressions of ALP, RUNX2 and Collagen type І were upregulated by 1% ECP‐CS (Figure 7C,F). According to the outcomes of Alizarin Red S staining and ALP staining, the number of osteoblasts and the size and degree of mineralized nodules were memorably improved when treated with 1% ECP‐CS compared with LPS group or 1% ECP‐BS (Figure 7D,E). These results indicated that ECP‐CS substantially attenuated the inhibitory role of LPS in differentiation and mineralization.

### 3.11 ECP reversed the inhibition of osteoblasts caused by LPS through LncRNA MALAT1/miR‐34a‐5p/SMAD2 axis

**FIGURE 8:**
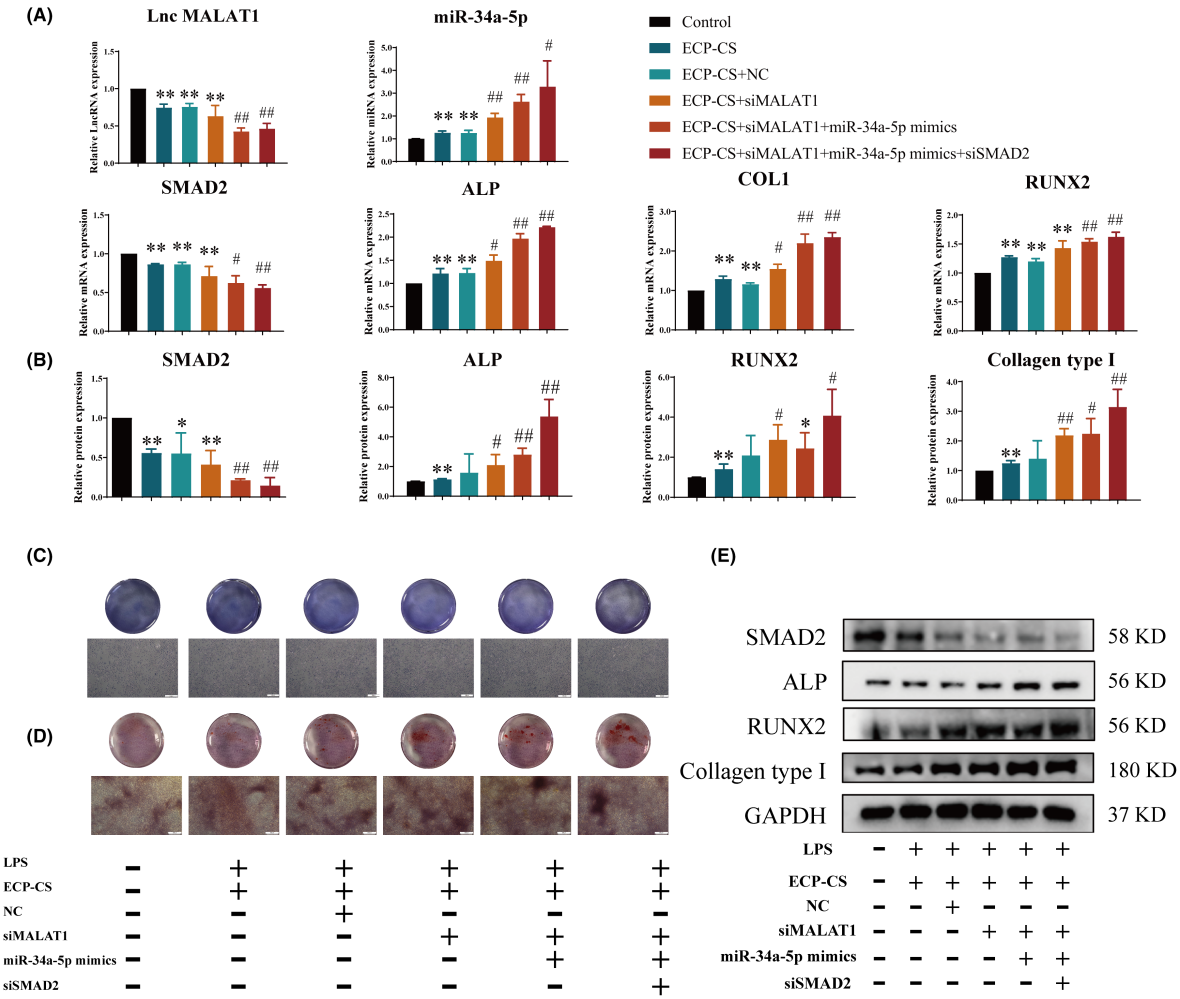
Verification of that ECP‐CS mediated LncRNA MALAT1 /miR‐34a‐5p /SMAD2 signalling pathway on osteoblasts. (A) qRT‐PCR was employed for detecting the gene expression of miR‐34a‐5p, SMAD2, ALP, RUNX2 and Collagen type І in osteoblasts. (B, E) Western Blot was employed for detecting the protein expression of total SMAD2, ALP, RUNX2 and Collagen type І. (C) The differentiation abilities of osteoblasts were observed by ALP staining (40×, scale bar = 500 μm). (D) The mineralization abilities of osteoblasts were observed by Alizarin red (40×, scale bar = 500 μm). (*p < 0.05, vs. Control; **p < 0.01, vs. Control; ^#^ *p* < 0.05, vs. ECP‐CS; ^##^ *p* < 0.01, vs. ECP‐CS, *n* = 3).

We utilized the same approach to verify whether ECP‐CS mediated MALAT1/miR‐34a‐5p/SMAD2 signal axis protects osteoblasts from LPS‐induced injury. According to the outcomes of qRT‐PCR, the gene expressions of LncRNA MALAT1 and SMAD2 in LPS + ECP‐CS + siMALAT1 group was significantly reduced, while the gene expressions of miR‐34a‐5p, ALP, RUNX2 and Collagen type І were increased compared with ECP‐CS group compared with LPS + ECP‐CS group (Figure 8A). At the same time, LPS + ECP‐CS + siMALAT1 treatment downregulated the protein expression of SMAD2 and upregulated the protein expressions of ALP, RUNX2 and Collagen type І (Figure 8B,E). The same conclusion was obtained for the Alizarin Red S staining and the ALP staining. LPS + ECP‐CS + siMALAT1 group could improve the number of cells and the extent of mineralized nodules (Figure 8C,D). After that, simultaneously transferring into siMALAT1 and miR‐34a‐5p mimics, the gene expressions of miR‐34a‐5p, SMAD2, ALP, RUNX2 and Collagen type І were grown and the gene expression of SMAD2 was declined memorably (Figure 8A). Meanwhile, the protein expressions of ALP, RUNX2 and Collagen type І were more improved, the protein expression of SMAD2 was further reduced (Figure 8B,E). Subsequently, when siMALAT1, miR‐34a‐5p mimics and siSMAD2 worked together, LPS + ECP‐CS + siMALAT1 + miR‐34a‐5p mimics + siSMAD2 group could have a better effect of the gene and protein expressions of SMAD2, ALP, RUNX2 and Collagen type І (Figure 8A,B,E). The outcomes of Alizarin Red S staining and ALP staining showed that the differentiation and mineralization abilities of osteoblasts in LPS+ ECP‐CS + siMALAT1 + miR‐34a‐5p mimics group were more improved than the LPS+ ECP‐CS + siMALAT1 + miR‐34a‐5p mimics +siSMAD2 group (Figure 8C,D). These results dropped a hint that LPS mediated the differentiation and mineralization of osteoblasts through LncRNA MALAT1/miR‐34a‐5p/SMAD2 signalling axis.

## 4 DISCUSSION

It is noteworthy that bone repair is a troublesome problem in IBDs. According to the TCM theory, tonifying kidney and strengthening bone medicine are the principal treatment methods in numerous bone related diseases.^23^ Both *Epimedium breviconu* Maxim (Epimedium) and *Curculigo orchioides* Gaertn (Curculigo orchioides), have the effects of nourishing kidney yang, strengthening bones and muscles and eliminating wind.^24^, ^25^ Pharmacological research has displayed that the flavonoids in Epimedium^9^, ^26^ or polysaccharides in Curculigo orchioides^27^, ^28^ could mediate osteoblasts' activities and then promote bone repair.

*Staphylococcus aureus* is the most studied bacteria in bone infection, which have been reported to stimulate bone resorption by secreting immune modulating proteins.^29^ Meanwhile, vancomycin has been considered as the ‘last resort’ treatment of infections caused by *Staphylococcus aureus* over the decades.^30^ Thus, in the present study, the tibias of rabbits were infected with *Staphylococcus aureus* for 4 weeks and then implanted Van‐CS to establish the IBDs model.^20^ The results showed that there was obvious bone loss in Model group and a defect cavity in Van‐CS + ECP group, which indicated that the IBDs model had been successfully constructed.

Transforming growth factor‐beta (TGF‐β)/bone morphogenic protein (BMP) signalling have widely recognized roles in bone formation and have two pathways: canonical SMAD‐dependent pathways^31^ and non‐canonical SMAD‐independent signalling pathway (such as MAPK).^32^ Researchers found that both of them converge on the RUNX2 gene to control cell differentiation.^33^ The coordinated activity of RUNX2 and TGF‐β/BMP‐activated SMADs is essential for skeleton formation. Our results showed that BV/TV, BMD and the protein expressions of BMP‐2 and RUNX2 in Van‐CS + ECP group were higher than those in Model and Van‐CS groups, which were consistent with the regulatory machinery of RUNX2 for skeletal gene expression^32^ and the antagonism of BMP2 and TGF‐β signalling during osteogenesis.^18^ These results suggested that ECP exert protective effects on bone repair in IBDs. These outcomes hinted that ECP could significantly increase the protein expressions of BMP‐2 and RUNX2 to facilitate bone repair in IBDs. Above all, the results suggested that ECP exert protective effects on bone repair in IBDs. However, there were still significant works to be done in investigating the absorbed constituents and molecular mechanisms.

It is well‐known that the active ingredients of Chinese Medicine exerted pharmacological effects through entering the bloodstream and transporting into various target organs and cells.^34^ Hence, UPLC‐QTRAP‐MS/MS method was employed to analysis the absorbed constituents of ECP. Six components were detected in ECP‐CS, including Curculigoside, Baohuoside I, Epimedin A/B, Icariin and Orcinol glucoside. Notably, Epimedin A/B and Icariin have been considered to be the hallmark compounds of the *Epimedium brevicornum maxim*, which have good antiosteoporotic activities through stimulating osteogenic differentiation and then accelerating the process of bone repair.^35^, ^36^, ^37^ Meanwhile, Baohuoside I is one of the metabolites of Icariin in vivo and also one of the main active flavonoid components of the *Epimedium brevicornum maxim*.^38^ Previous studies have shown that Curculigoside, the effective substance of Curculigo orchioides, could attenuate wear particle‐induced periprosthetic osteolysis by promoting osteoblastic MC3T3‐E1 cell differentiation and inhibiting osteoclast BMSC formation.^39^ It is important to note that there is currently a lack of literature on the correlation between Orcinol glucoside and bone. Our study revealed that Orcinol glucoside was the highest concentration of the absorbed constituents. As a result, we intend to pursue further research on Orcinol glucoside with an aim to explore its potential as a novel therapeutic agent for bone regeneration.

To explore the mechanisms of ECP in IBDs, the network pharmacology approaches were performed. The results showed that these six absorbed constituents were concentrated in TGF‐β/SMADs signalling pathway^40^ for bone repair and inflammatory, which were the key to the treatment of IBDs.^41^ Research has proven that TGF‐β/SMADs signalling pathway could affect the activities of osteoblasts, which exert a key effect on the control of bone remodelling.^42^ Combining with previous literature searches and extensive literature, it was ultimately predicted that these six components in ECP extract might promote osteoblasts' activities to stimulate the bone repair in infected bone defects through the TGF‐β/SMADs signalling pathway.

**FIGURE 9:**
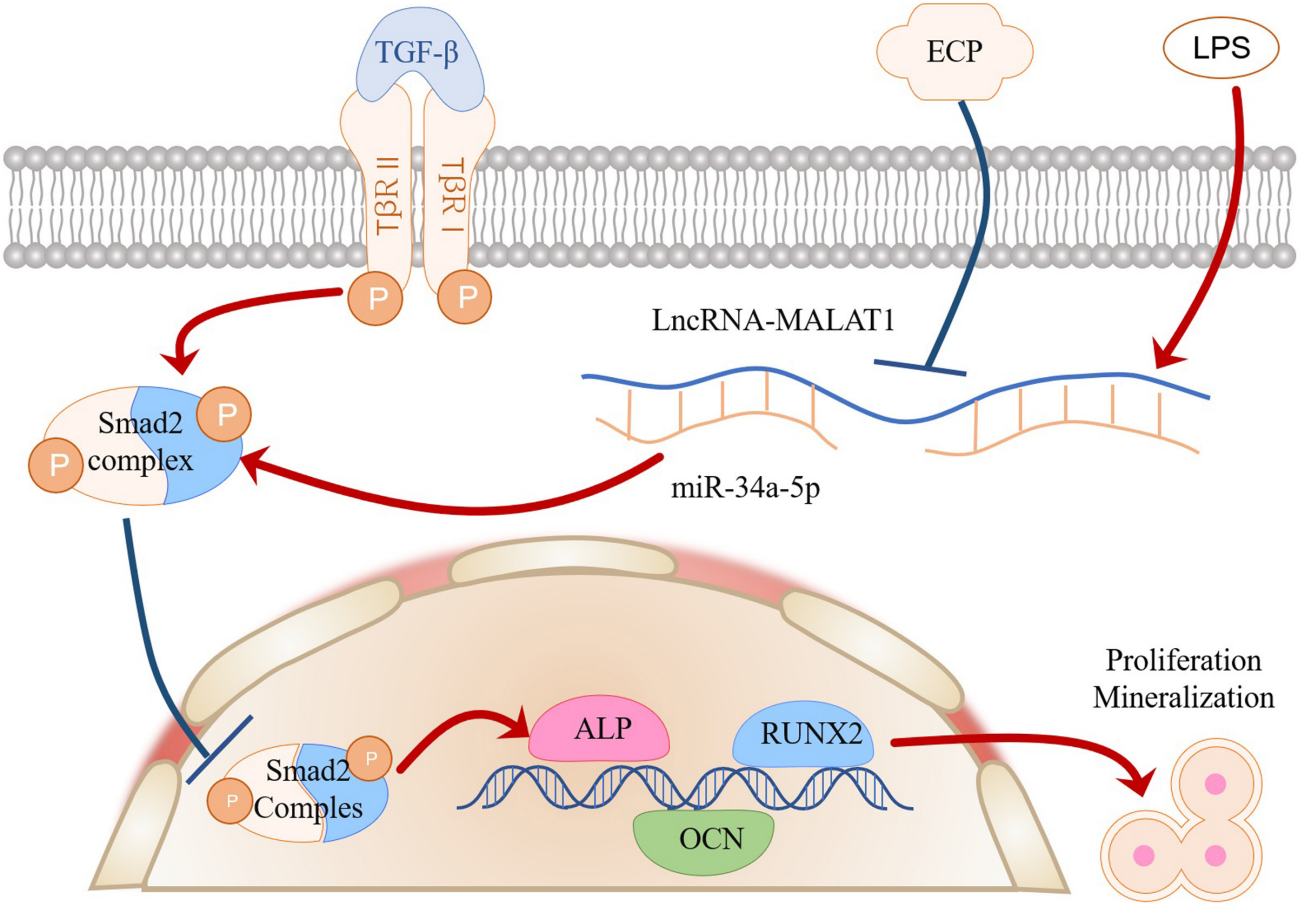
The mechanism of Epimedium‐Curculigo herb pair on bone repair after IBDs.

Previous studies have suggested that LncRNA MALAT1 promotes osteoblast differentiation by competing with microRNAs (miRNAs), that is a ‘miRNA sponge’, in cell culture.^43^, ^44^, ^45^ Indeed, the reported role for LncRNA MALAT1 in mediating osteoblast differentiation has been linked to the sponging of several miRNAs including miR‐204,^46^ miR‐30^44^ and miR‐143.^47^ In our study, the luciferase reporter assay substantiated that LncRNA MALAT1 modulate SMAD2 expression via being a miR‐34a‐5p sponge. According to the outcomes of qRT‐PCR and Western Blot, when osteoblasts were transferred into siMALAT1 or siSMAD2, it could significantly promote differentiation and mineralization of osteoblasts during miR‐34a‐5p inhibitor co‐culture. Our results are in line with previous studies showing that LncRNA MALAT1 participates in osteogenic processes by binding miRNAs. Inflammatory bone diseases, including osteomyelitis and periodontitis, were characterized by osteoblast dysfunction, which contributes significantly to inflammation‐induced bone degradation^48^ Lipopolysaccharide (LPS), a critical inducer of bone erosion associated with gram‐negative bacteria, hinders the differentiation and mineralization of osteoblasts. Here in our study, we also validated that LPS regulated the differentiation and mineralization of osteoblasts by mediating the LncRNA MALAT1 /miR‐34a‐5p/ SMAD2 signalling pathway. When osteoblasts were transferred into siMALAT1, siSMAD2 or miR‐34a‐5p mimics, it could also substantially reverse the inhibition of normal osteoblasts activities during LPS culture. These results dropped a hint that LPS mediates the differentiation and mineralization of osteoblasts through the LncRNA MALAT1/miR‐34a‐5p/SMAD2 signalling axis. Furthermore, research indicates that the expression of LncRNA MALAT1 is decreased during the process of osteoclast differentiation, leading to bone resorption, ultimately contributing to low bone mineral density and the development of osteoporosis.^49^ It is hypothesized that LncRNA MALAT1 plays a significant role in bone repair by promoting osteoblast function and inhibiting osteoclast activity, thereby serving as a potential therapeutic target for the prevention and treatment of osteoporosis and bone defects. Finally, we validated the efficacy of ECP‐CS on osteoblasts. The expressions of LncRNA MALAT1 and SMAD2 were blocked, while the expressions of miR‐34a‐5p, ALP, RUNX2 and Collagen type І were elevated dramatically, when ECP‐CS intervened in LPS‐induced osteoblasts. Meanwhile, knocking down LncRNA MALAT1 and SMAD2, or enhancing miR‐34a‐5p in osteoblasts could significantly promote the osteoblasts' differentiation and mineralization abilities in ECP‐CS‐LPS‐cultured osteoblasts. In summary, ECP could alleviate the damage caused by LPS through the LncRNA MALAT1/miR‐34a‐5p/SMAD2 axis in osteoblasts (as shown in Figure 9). The completion of this study provided a new strategy and some novel therapeutic targets for the clinical treatment of IBDs.

## 5 CONCLUSION

Our study confirmed that Epimedium‐Curculigo herb pair could stimulate the differentiation and mineralization of osteoblasts by regulating LncRNA MALAT1/miR‐34a‐5p/SMAD2 signalling pathway in infected bone defects, therefor, have the enhancing effect of bone healing.

## AUTHOR CONTRIBUTIONS

**Maomao Miao:** Conceptualization (equal); data curation (equal); formal analysis (equal); methodology (equal); writing – original draft (equal). **Mengying Li:** Data curation (equal); methodology (equal). **Yunjie Sheng:** Data curation (equal). **Peijian Tong:** Funding acquisition (equal); project administration (equal). **Yang Zhang:** Conceptualization (equal); funding acquisition (equal); project administration (equal); writing – review and editing (equal). **Dan Shou:** Funding acquisition (equal); project administration (equal).

## FUNDING INFORMATION

This study was supported by the Zhejiang Province ‘High‐level Talents Special Support Program’‐Science and Technology innovation Leading talents project (grant no. 2022R52031), Zhejiang Provincial Natural Science Foundation of China (grant no. LD22C060002), Zhejiang Provincial Medical and Health Science and Technology Fund (grant no. 2021KY112), Zhejiang Provincial Traditional Chinese Medicine Science and Technology Fund (grant no. 2021ZA033), Research Project of Zhejiang Chinese Medical University (grant no. 2023JKZKTS34) and the Project of Chunyan Special Fund for Chinese Medicine Development of Zhejiang Chinese Medical University under (grant no. CY202305).

## CONFLICT OF INTEREST STATEMENT

The authors declare that they have no conflict of interest.

## Supporting information


Figures S1–S3.

Tables S1–S8.

## Data Availability

The original data supporting the conclusions of this article will be provided by the authors, without undue reservation.
