# Supplementary material for: Epimedium‐Curculigo herb pair enhances bone repair with infected bone defects and regulates osteoblasts through LncRNA MALAT1/miR‐34a‐5p/SMAD2 axis
Source: J Cell Mol Med. 2024 Jul 10;28(13):e18527. doi: 10.1111/jcmm.18527 (PMC11234645; doi:10.1111/jcmm.18527)
Supplement: Supplementary file 1 — Figures S1–S3. [file JCMM-28-e18527-s002.pdf]

Supplementary Figure

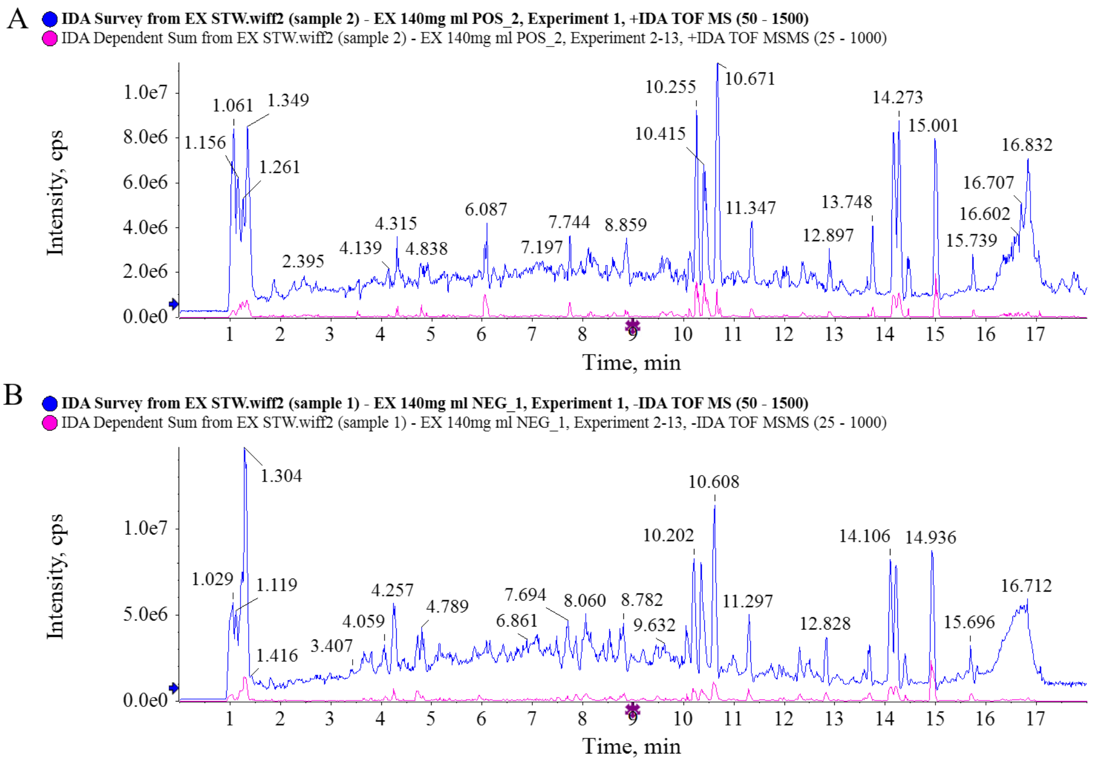

Figure S1 UPLC-QTOF-MS/MS Spectrogram of ECP Extract

(A: TIC of ECP in positive ion mode; B: TIC of ECP in negative ion mode)

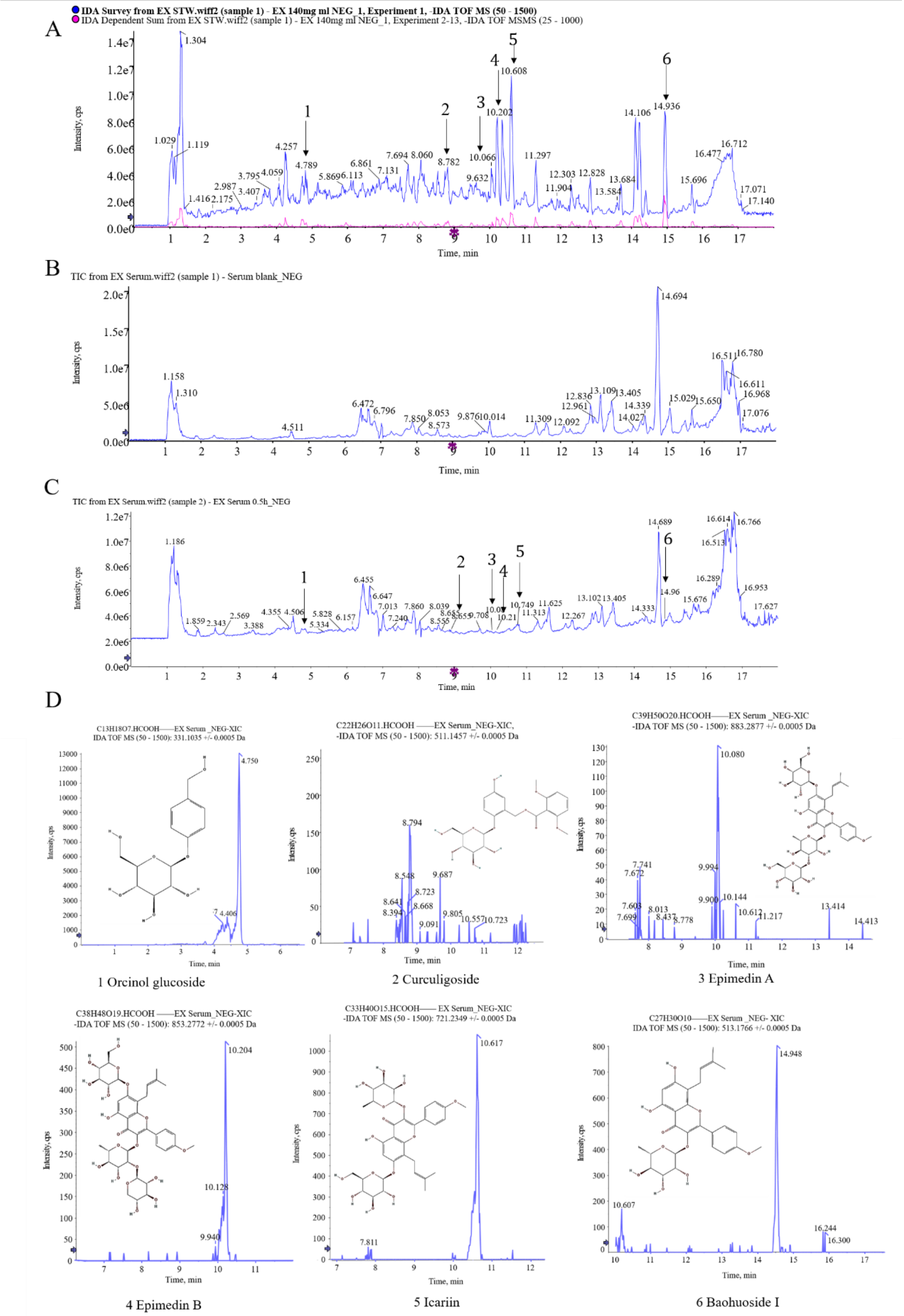

Figure S2 UPLC-QTOF-MS/MS mass spectrometry of ECP, BM and ECP-MS (A: TIC of ECP extract in negative ion mode; B: TIC of blank serum in negative ion mode; C: TIC of ECP-MS in negative ion mode; D: Extracted ion chromatography (XIC) of six components in ECP-CS)

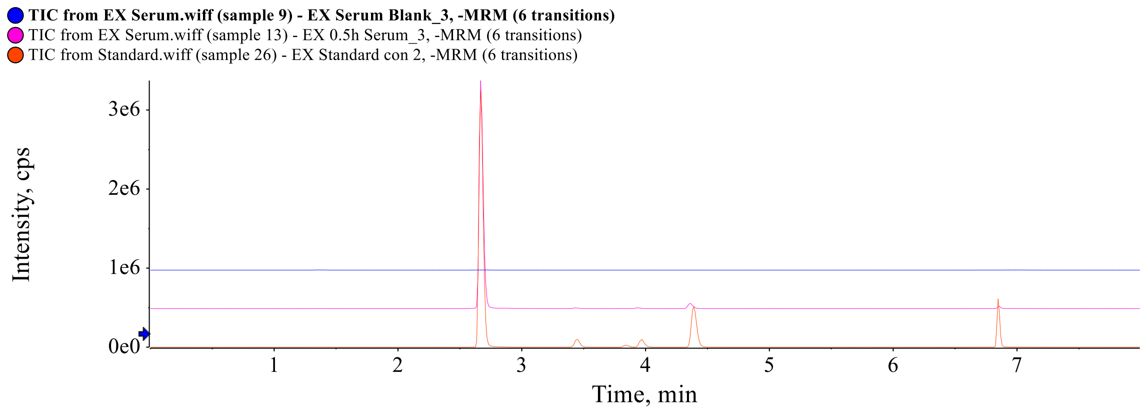

Figure S3 Selectivity of UPLC-QTRAP-MS/MS method
