# Supplementary material for: Epimedium‐Curculigo herb pair enhances bone repair with infected bone defects and regulates osteoblasts through LncRNA MALAT1/miR‐34a‐5p/SMAD2 axis
Source: J Cell Mol Med. 2024 Jul 10;28(13):e18527. doi: 10.1111/jcmm.18527 (PMC11234645; doi:10.1111/jcmm.18527)
Supplement: Supplementary file 2 — Tables S1–S8. [file JCMM-28-e18527-s001.docx]

**Supplementary Table**

Table S1 MRM parameters of components detected in ECP-CS

| Index | Component Name | Formula | Precursor ion (Da) | Fragment ion (Da) | Retention Time | Adduct |
| --- | --- | --- | --- | --- | --- | --- |
| 1 | Baohuoside I | C_27_H_30_O_10_ | 513.2 | 366.1 | 6.85 | [M-H]^-^ |
| 2 | Icarrin | C_33_H_40_O_15_ | 675.2 | 513.1 | 4.35 | [M-H]^-^ |
| 3 | Epimedin B | C_38_H_48_O_19_ | 807.2 | 645.1 | 3.94 | [M-H]^-^ |
| 4 | Orcinol glucosid | C_13_H_18_O_7_ | 284.9 | 123.1 | 2.67 | [M-H]^-^ |
| 5 | Curculigoside | C_22_H_26_O_11_ | 465.1 | 283 | 3.44 | [M-H]^-^ |
| 6 | A Epimedin A | C_39_H_50_O_20_ | 837.2 | 675.3 | 3.81 | [M-H]^-^ |

Table S2 siRNA synthetic chain sequence

| ncRNA | Sense (5’-3’) | Anti-sense (5’-3’) |
| --- | --- | --- |
| siMALAT1 | GGCCAGAGGUUGAAGUUAATT | UUAACUUCAACCUCUGGCCTT |
| miR-34a-5p mimics | UGGCAGUGUCUUAGCUGGUUGU | AACCAGCUAAGACACUGCCAUU |
| miR-34a-5p inhibitor | ACAACCAGCUAAGACACUGCCA |  |
| siSMAD2 | GCCUAAGUGAUAGUGCGAUTT | AUCGCACUAUCACUUAGGCTT |
| Negative Control | UUCUCCGAACGUGUCACGUTT | ACGUGACACGUUCGGAGAATT |
| Inhibitor NC | CAGUACUUUUGUGUAGUACAA |  |

Table S3 Primers sequences for qRT‑PCR

| Gene | Forward (5’-3’) | Reverse (5’-3’) |
| --- | --- | --- |
| LncMALAT1 | GTTACCAGCCCAAACCTCAA | CGATGTGGCAGAGAAATCAC |
| miR-34a-5p | ACTTCCTCTGGCAGTGTCTTAGC | ATCCAGTGCAGGGTCCGAGG |
| U6 | CTCGCTTCGGCAGCACATATACT | ACGCTTCACGAATTTGCGTGTC |
| SMAD2 | ATGTCGTCCATCTTGCCATTCACTC | TCCATTCTGCTCTCCACCACCTG |
| ALP | CGGCGTCCATGAGCAGAACTAC | CAGGCACAGTGGTCAAGGTTGG |
| RUNX2 | GATGATGACACTGCCACCTCTGAC | TGAGGGATGAAATGCTTGGGAACTG |
| Collagen type І | GACAGGCGAACAAGGTGACAGAG | CAGGAGAACCAGGAGAACCAGGAG |
| β-actin | CTGAGAGGGAAATCGTGCGTGAC | ACCGCTCGTTGCCAATAGTGATG |

Table S4 The positive spectral peaks of ECP extract under Total ion chromatogram (TIC)

| Index | Component Name | Retention Time | Formula | Precursor Mass | Found At Mass | Mass Error (ppm) | Adduction |
| --- | --- | --- | --- | --- | --- | --- | --- |
| 1 | Stachydrine | 1.35 | C_7_H_13_NO_2_ | 144.1019 | 144.1017 | -1.5 | [M+H]^+^ |
| 2 | Higenamine | 4.57 | C_16_H_17_NO_3_ | 272.1281 | 272.128 | -0.4 | [M+H]^+^ |
| 3 | Puerarin | 5.76 | C_21_H_20_O_9_ | 417.118 | 417.118 | 0 | [M+H]^+^ |
| 4 | Albiflorin | 6.46 | C_23_H_28_O_11_ | 481.1704 | 481.1706 | 0.3 | [M+H]^+^ |
| 5 | Paeoniflorin +NH3 | 6.83 | C_23_H_28_O_11_.NH_3_ | 498.197 | 498.1971 | 0.3 | [M+H]^+^ |
| 6 | Kaempferitrin | 7.42 | C_27_H_30_O_14_ | 579.1708 | 579.1707 | -0.2 | [M+H]^+^ |
| 7 | Liquiritigenin | 7.56 | C_15_H_12_O_4_ | 257.0808 | 257.081 | 0.6 | [M+H]^+^ |
| 8 | Scutellarin | 7.67 | C_21_H_18_O_12_ | 463.0871 | 463.0874 | 0.6 | [M+H]^+^ |
| 9 | Ononin | 9.63 | C_22_H_22_O_9_ | 431.1337 | 431.1339 | 0.7 | [M+H]^+^ |
| 10 | Daidzein | 9.88 | C_15_H_10_O_4_ | 255.0652 | 255.0652 | 0.1 | [M+H]^+^ |
| 11 | Epimedin A | 10.13 | C_39_H_50_O_20_ | 839.2968 | 839.2961 | -0.8 | [M+H]^+^ |
| 12 | Epimedin B | 10.25 | C_38_H_48_O_19_ | 809.2863 | 809.2849 | -1.6 | [M+H]^+^ |
| 13 | Epimedin C | 10.42 | C_39_H_50_O_19_ | 823.3019 | 823.3012 | -0.9 | [M+H]^+^ |
| 14 | Icarrin | 10.67 | C_33_H_40_O_15_ | 677.244 | 677.243 | -1.5 | [M+H]^+^ |
| 15 | Wogonin 7-O-glucuronide | 10.99 | C_22_H_20_O_11_ | 461.1078 | 461.1077 | -0.3 | [M+H]^+^ |
| 16 | Icaritin | 11.13 | C_21_H_22_O_7_ | 387.1438 | 387.1438 | -0.1 | [M+H]^+^ |
| 17 | Baicalein | 12.49 | C_15_H_10_O_5_ | 271.0601 | 271.0601 | -0.1 | [M+H]^+^ |
| 18 | Formononetin | 13.1 | C_16_H_12_O_4_ | 269.0808 | 269.0807 | -0.6 | [M+H]^+^ |
| 19 | Glycyrrhizic acid | 13.37 | C_42_H_62_O_16_ | 823.4111 | 823.4104 | -0.8 | [M+H]^+^ |
| 20 | Wogonin | 14.43 | C_16_H_12_O_5_ | 285.0757 | 285.0757 | -0.3 | [M+H]^+^ |
| 21 | Baohuoside I | 15 | C_27_H_30_O_10_ | 515.1912 | 515.1906 | -1 | [M+H]^+^ |

Table S5 The negative spectral peaks of ECP extract under Total ion chromatogram (TIC)

| Index | Component Name | Retention Time | Formula | Precursor Mass | Found At Mass | Mass Error (ppm) | Adduction |
| --- | --- | --- | --- | --- | --- | --- | --- |
| 1 | Quinic acid | 1.3 | C_7_H_12_O_6_ | 191.0561 | 191.0559 | -0.9 | [M-H]^-^ |
| 2 | Gallic acid | 2.84 | C_7_H_6_O_5_ | 169.0142 | 169.014 | -1.4 | [M-H]^-^ |
| 3 | Protocatechuic acid | 4.09 | C_7_H_6_O_4_ | 153.0193 | 153.0191 | -1.3 | [M-H]^-^ |
| 4 | Loganic acid | 4.61 | C_16_H_24_O_10_ | 375.1297 | 375.1293 | -1.1 | [M-H]^-^ |
| 5 | Orcinol glucosid | 4.73 | C_13_H_18_O_7_ | 285.098 | 285.0975 | -1.8 | [M-H]^-^ |
| 6 | Catechin | 5.22 | C_15_H_14_O_6_ | 289.0718 | 289.0711 | -2.3 | [M-H]^-^ |
| 7 | Puerarin | 5.71 | C_21_H_20_O_9_ | 415.1035 | 415.1028 | -1.5 | [M-H]^-^ |
| 8 | Caffeic acid | 5.81 | C_9_H_8_O_4_ | 179.035 | 179.0346 | -1.9 | [M-H]^-^ |
| 9 | Kaempferitrin | 7.37 | C_27_H_30_O_14_ | 577.1563 | 577.1553 | -1.7 | [M-H]^-^ |
| 10 | Quercitrin | 8.2 | C_21_H_20_O_11_ | 447.0933 | 447.0923 | -2.2 | [M-H]^-^ |
| 11 | Naringin | 8.26 | C_27_H_32_O_14_ | 579.1719 | 579.171 | -1.6 | [M-H]^-^ |
| 12 | Hesperidin | 8.48 | C_28_H_34_O_15_ | 609.1825 | 609.1815 | -1.6 | [M-H]^-^ |
| 13 | Curculigoside +HCOOH | 8.76 | C_22_H_26_O_11_.HCOOH | 511.1457 | 511.1449 | -1.5 | [M-H]^-^ |
| 14 | Timosaponin BII +HCOOH | 9.32 | C_45_H_76_O_19_.HCOOH | 965.4963 | 965.4953 | -1 | [M-H]^-^ |
| 15 | Daidzein | 9.79 | C_15_H_10_O_4_ | 253.0506 | 253.0499 | -3 | [M-H]^-^ |
| 16 | Epimedin A | 10.07 | C_39_H_50_O_20_ | 837.2823 | 837.2806 | -2 | [M-H]^-^ |
| 17 | Epimedin B +HCOOH | 10.21 | C_38_H_48_O_19_.HCOOH | 853.2772 | 853.2753 | -2.2 | [M-H]^-^ |
| 18 | Epimedin C | 10.39 | C_39_H_50_O_19_ | 821.2874 | 821.2864 | -1.1 | [M-H]^-^ |
| 19 | Icarrin +HCOOH | 10.61 | C_33_H_40_O_15_.HCOOH | 721.2349 | 721.2338 | -1.6 | [M-H]^-^ |
| 20 | Wogonin 7-O-glucuronide | 10.91 | C_22_H_20_O_11_ | 459.0933 | 459.0924 | -2 | [M-H]^-^ |
| 21 | Naringenin | 11.49 | C_15_H_12_O_5_ | 271.0612 | 271.0608 | -1.5 | [M-H]^-^ |
| 22 | Baicalein | 12.41 | C_15_H_10_O_5_ | 269.0455 | 269.0449 | -2.3 | [M-H]^-^ |
| 23 | Ginsenoside Rh1 +HCOOH | 12.44 | C_36_H_62_O_9_.HCOOH | 683.4376 | 683.4361 | -2.2 | [M-H]^-^ |
| 24 | Ginsenoside Rg2 | 12.49 | C_42_H_72_O_13_ | 783.49 | 783.4884 | -2.1 | [M-H]^-^ |
| 25 | Glycyrrhizic acid | 13.32 | C_42_H_62_O_16_ | 821.3965 | 821.3949 | -2 | [M-H]^-^ |
| 26 | Baohuoside I | 14.94 | C_27_H_30_O_10_ | 513.1766 | 513.1752 | -2.8 | [M-H]^-^ |

Table S6 The UPLC-QTOF-MS/MS analysis of main components in ECP extract

| Index | Component Name | Retention Time | Formula | Precursor Mass | Found At Mass | Mass Error (ppm) | Adduction |
| --- | --- | --- | --- | --- | --- | --- | --- |
| 1 | Quinic acid | 1.3 | C_7_H_12_O_6_ | 191.0561 | 191.0559 | -0.9 | [M-H]^-^ |
| 2 | Stachydrine | 1.35 | C_7_H_13_NO_2_ | 144.1019 | 144.1017 | -1.5 | [M+H]^+^ |
| 3 | Gallic acid | 2.84 | C_7_H_6_O_5_ | 169.0142 | 169.014 | -1.4 | [M-H]^-^ |
| 4 | Protocatechuic acid | 4.09 | C_7_H_6_O_4_ | 153.0193 | 153.0191 | -1.3 | [M-H]^-^ |
| 5 | Higenamine | 4.57 | C_16_H_17_NO_3_ | 272.1281 | 272.128 | -0.4 | [M+H]^+^ |
| 6 | Loganic acid | 4.61 | C_16_H_24_O_10_ | 375.1297 | 375.1293 | -1.1 | [M-H]^-^ |
| 7 | Orcinol glucosid | 4.73 | C_13_H_18_O_7_ | 285.098 | 285.0975 | -1.8 | [M-H]^-^ |
| 8 | Catechin | 5.22 | C_15_H_14_O_6_ | 289.0718 | 289.0711 | -2.3 | [M-H]^-^ |
| 9 | Puerarin | 5.71 | C_21_H_20_O_9_ | 415.1035 | 415.1028 | -1.5 | [M-H]^-^ |
| 10 | Caffeic acid | 5.81 | C_9_H_8_O_4_ | 179.035 | 179.0346 | -1.9 | [M-H]^-^ |
| 11 | Albiflorin | 6.46 | C_23_H_28_O_11_ | 481.1704 | 481.1706 | 0.3 | [M+H]^+^ |
| 12 | Paeoniflorin +NH3 | 6.83 | C_23_H_28_O_11_.NH_3_ | 498.197 | 498.1971 | 0.3 | [M+H]^+^ |
| 13 | Kaempferitrin | 7.37 | C_27_H_30_O_14_ | 577.1563 | 577.1553 | -1.7 | [M-H]^-^ |
| 14 | Scutellarin | 7.67 | C_21_H_18_O_12_ | 463.0871 | 463.0874 | 0.6 | [M+H]^+^ |
| 15 | Quercitrin | 8.2 | C_21_H_20_O_11_ | 447.0933 | 447.0923 | -2.2 | [M-H]^-^ |
| 16 | Naringin | 8.26 | C_27_H_32_O_14_ | 579.1719 | 579.171 | -1.6 | [M-H]^-^ |
| 17 | Hesperidin | 8.48 | C_28_H_34_O_15_ | 609.1825 | 609.1815 | -1.6 | [M-H]^-^ |
| 18 | Curculigoside +HCOOH | 8.76 | C_22_H_26_O_11_.HCOOH | 511.1457 | 511.1449 | -1.5 | [M-H]^-^ |
| 19 | Timosaponin BII +HCOOH | 9.32 | C_45_H_76_O_19_.HCOOH | 965.4963 | 965.4953 | -1 | [M-H]^-^ |
| 20 | Ononin | 9.63 | C_22_H_22_O_9_ | 431.1337 | 431.1339 | 0.7 | [M+H]^+^ |
| 21 | Daidzein | 9.79 | C_15_H_10_O_4_ | 253.0506 | 253.0499 | -3 | [M-H]^-^ |
| 22 | Epimedin A | 10.07 | C_39_H_50_O_20_ | 837.2823 | 837.2806 | -2 | [M-H]^-^ |
| 23 | Epimedin B +HCOOH | 10.21 | C_38_H_48_O_19_.HCOOH | 853.2772 | 853.2753 | -2.2 | [M-H]^-^ |
| 24 | Epimedin C | 10.39 | C_39_H_50_O_19_ | 821.2874 | 821.2864 | -1.1 | [M-H]^-^ |
| 25 | Icarrin +HCOOH | 10.61 | C_33_H_40_O_15_.HCOOH | 721.2349 | 721.2338 | -1.6 | [M-H]^-^ |
| 26 | Wogonin 7-O-glucuronide | 10.91 | C_22_H_20_O_11_ | 459.0933 | 459.0924 | -2 | [M-H]^-^ |
| 27 | Icaritin | 11.13 | C_21_H_22_O_7_ | 387.1438 | 387.1438 | -0.1 | [M+H]^+^ |
| 28 | Naringenin | 11.49 | C_15_H_12_O_5_ | 271.0612 | 271.0608 | -1.5 | [M-H]^-^ |
| 29 | Baicalein | 12.41 | C_15_H_10_O_5_ | 269.0455 | 269.0449 | -2.3 | [M-H]^-^ |
| 30 | Ginsenoside Rh1 +HCOOH | 12.44 | C_36_H_62_O_9_.HCOOH | 683.4376 | 683.4361 | -2.2 | [M-H]^-^ |
| 31 | Ginsenoside Rg2 | 12.49 | C_42_H_72_O_13_ | 783.49 | 783.4884 | -2.1 | [M-H]^-^ |
| 32 | Formononetin | 13.1 | C_16_H_12_O_4_ | 269.0808 | 269.0807 | -0.6 | [M+H]^+^ |
| 33 | Glycyrrhizic acid | 13.32 | C_42_H_62_O_16_ | 821.3965 | 821.3949 | -2 | [M-H]^-^ |
| 34 | Baohuoside I | 14.94 | C_27_H_30_O_10_ | 513.1766 | 513.1752 | -2.8 | [M-H]^-^ |

Table S7 UPLC-QTOF-MS/MS mass spectrometry analysis the main components absorbed into blood in ECP extract

| Index | Component Name | Retention Time /min | Formula | Precursor Mass /m.z^-1^ | Found At Mass /m.z^-1^ | Mass Error /ppm | Fragment ions/m/z  [MS^1^] | Fragment ions/m/z  [MS^2^] |
| --- | --- | --- | --- | --- | --- | --- | --- | --- |
| 1 | Orcinol glucosid | 4.75 | C_13_H_18_O_7_ | 331.1035 | 331.103 | -1.3 | 289.0263；288.0599；287.0357； | 157.0077；123.0448； 123.0623；81.0341；43.0168; |
| 2 | Curculigoside | 8.79 | C_22_H_26_O_11_ | 511.1457 | 511.147 | 2.6 | 515.2870；514.2838；514.2338； | 431.2114；283.0794；137.0590 |
| 3 | Epimedin A | 10.08 | C_39_H_50_O_20_ | 883.2877 | 883.2848 | -3.3 | 841.2996；840.2915；839.2869；838.2869；837.3737； | 837.4220；675.2889；675.2321；675.3339；675.1656；513.1855；366.1099；323.0937； |
| 4 | Epimedin B | 10.21 | C_38_H_48_O_19_ | 853.2772 | 853.2754 | -2.1 | 812.1158；811.2873；810.2809；809.2794；808.2764； | 645.1643；645.2202；645.2934；352.0934；366.1108；366.1324；280.0416； |
| 5 | Icariin | 10.61 | C_33_H_40_O_15_ | 721.2349 | 721.2342 | -1 | 679.2518；678.2471；677.2326；676.2330；675.3067；675.2296； | 675.2344；675.2086；675.1896；513.1819；513.1709；382.1235；367.1301；366.1142；382.1082；312.0719 |
| 6 | Baohuoside I | 14.95 | C_27_H_30_O_10_ | 513.1766 | 513.1757 | -1.7 | 517.1852；516.1882；515.1834；514.1807；513.2494； | 513.1798；513.1101；367.1201；366.1125；351.0889；323.0921；311.0574；295.0625；293.1197；217.0521； |

Table S8 The validation of UPLC-QTRAP-MS/MS method

| Component Name | Concentration | Accuracy(%) | Precision(RSD%) | Stability | | Extraction Recovery(%) | Matrix Eﬀect(%) |
| --- | --- | --- | --- | --- | --- | --- | --- |
|  |  |  |  | mean | Percent CV |  |  |
| Baohuoside I | 60 | 96.28 | 2.94 | 56.83 | 3.87 | 92.95 | 91.57 |
|  | 20 | 96.25 | 3.20 |  |  | 93.12 | 93.70 |
|  | 1.24 | 94.35 | 4.42 | 1.13 | 5.31 | 104.76 | 95.66 |
| Icariin | 210 | 91.38 | 1.92 | 193.42 | 3.13 | 89.76 | 87.90 |
|  | 42 | 93.67 | 0.89 |  |  | 89.58 | 93.10 |
|  | 1.1 | N/A | 5.07 | N/A | N/A | N/A | N/A |
| Epimedin B | 240 | 92.29 | 1.99 | 218.83 | 2.54 | 86.68 | 87.53 |
|  | 60 | 93.05 | 1.39 |  |  | 90.40 | 92.80 |
|  | 0.63 | 94.76 | 4.39 | 0.57 | 2.95 | 96.70 | 94.98 |
| Orcinol glucosid | 3000 | 105.07 | 2.79 | 3070.33 | 2.56 | 91.66 | 100.40 |
|  | 900 | 91.18 | 3.90 |  |  | 93.44 | 89.40 |
|  | 62.5 | 105.26 | 3.83 | 63.70 | 4.13 | 95.95 | 97.97 |
| Curculigoside | 30 | 101.23 | 3.55 | 32.86 | 3.92 | 92.17 | 92.90 |
|  | 10 | 102.70 | 4.54 |  |  | 95.97 | 97.50 |
|  | 1.25 | 106.96 | 4.28 | 1.32 | 1.32 | 98.69 | 98.84 |
| Epimedin A | 30 | 93.03 | 4.21 | 28.11 | 3.05 | 87.75 | 88.84 |
|  | 10 | 99.11 | 3.24 |  |  | 91.81 | 100.00 |
|  | 1.25 | 100.64 | 3.65 | 1.27 | 3.67 | 96.77 | 94.38 |
